# Attenuation of Choroidal Neovascularization by Histone Deacetylase Inhibitor

**DOI:** 10.1371/journal.pone.0120587

**Published:** 2015-03-25

**Authors:** Nymph Chan, Shikun He, Christine K. Spee, Keijiro Ishikawa, David R. Hinton

**Affiliations:** 1 Department of Pathology, Keck School of Medicine of the University of Southern California, Los Angeles, CA, United States of America; 2 Department of Ophthalmology, Keck School of Medicine of the University of Southern California, Los Angeles, CA, United States of America; 3 Doheny Eye Institute, Los Angeles, CA, United States of America; Indiana University College of Medicine, UNITED STATES

## Abstract

Choroidal neovascularization (CNV) is a blinding complication of age-related macular degeneration that manifests as the growth of immature choroidal blood vessels through Bruch’s membrane, where they can leak fluid or hemorrhage under the retina. Here, we demonstrate that the histone deacetylase inhibitor (HDACi) trichostatin A (TSA) can down-regulate the pro-angiogenic hypoxia-inducible factor-1α and vascular endothelial growth factor (VEGF), and up-regulate the anti-angiogenic and neuro-protective pigment epithelium derived factor in human retinal pigment epithelial (RPE) cells. Most strikingly, TSA markedly down-regulates the expression of VEGF receptor-2 in human vascular endothelial cells and, thus, can knock down pro-angiogenic cell signaling. Additionally, TSA suppresses CNV-associated wound healing response and RPE epithelial-mesenchymal transdifferentiation. In the laser-induced model of CNV using C57Bl/6 mice, systemic administration of TSA significantly reduces fluorescein leakage and the size of CNV lesions at post—laser days 7 and 14 as well as the immunohistochemical expression of VEGF, VEGFR2, and smooth muscle actin in CNV lesions at post-laser day 7. This report suggests that TSA, and possibly HDACi’s in general, should be further evaluated for their therapeutic potential for the treatment of CNV.

## Introduction

Choroidal neovascularization (CNV) is a serious blinding complication of the exudative form of age-related macular degeneration (AMD) [1]. CNV, defined as the pathological growth of immature choroidal blood vessels under the retinal pigment epithelium (RPE) and/or in the subretinal space, is associated with an imbalance between pro-angiogenic and anti-angiogenic factors [1], favoring a pro-angiogenic environment in the context of a wound healing response [2–11].

Many growth factors regulate CNV formation, including vascular endothelial growth factor (VEGF), angiopoietin 1 and 2, transforming growth factor-β (TGF-β), and pigment epithelium derived factor (PEDF) [12]. The expression of these growth factors can be regulated by hypoxia, ischemia, or inflammation [13], which is a wound healing response that involves inflammatory cells, blood vessel formation, epithelial-mesenchymal transdifferentiation (EMT) of the RPE [14], and fibrosis [15]. TGF-β is the major promoting factor of EMT and fibrosis [16], and is expressed in human RPE cells [17] and experimental rat CNV membranes [18]. TGF-β also induces VEGF expression in RPE cells and choroidal endothelial cells (CECs) and enhances CNV progression [19].

Located at the outer aspect of the retina, the RPE layer is in close proximity to the choroidal vessels, separated only by the Bruch’s membrane [20]. RPE cells are normally mitotically quiescent [21] while producing growth factors to maintain the viability of the choroidal endothelium [22–25] and trophic and metabolic support for the photoreceptors [26, 27]. When rabbits were injected with a RPE-specific toxin, sodium iodate, the choroid underwent atrophy in areas with RPE cell loss [22]. Further, the choroiocapillaris was reduced in areas with atrophic RPE in patients with geographic atrophy, whereas CNV lesions were associated with RPE cells, implying that choroidal vessel growth relies on the growth factors produced by RPE, [28] and the death of activated RPE cells at the end stage of CNV is related to the regression of choroidal angiogenesis.

In hypoxia, angiogenesis is regulated by the transcription factor hypoxia inducible factor 1 (HIF-1). Under hypoxic conditions, the stabilized oxygen-labile HIF-1α subunit binds with the constitutively expressed HIF-1β subunit and translocates to the nucleus to activate gene expression. HIF-1 recognizes the hypoxia-responsive element in the promoter of VEGF and mediates its expression. [20, 29] VEGF is expressed in RPE cells *in vitro* [2, 7] and *in vivo* [3–6, 8]. It promotes the survival, proliferation, and motility of endothelial cells (ECs), and regulates the structure of the vasculature. [30] Together with its cell surface receptor, VEGF receptor 2 (VEGFR2), it is highly expressed in cells in CNV lesions [30]. Overexpressed VEGF promotes retinal neovascularization in transgenic mice, and its enhanced production had been demonstrated in mouse CNV models, [13] a well-established laser-induced CNV model using C57Bl/6 mice that mimics many aspects of the pathology of human CNV [31]. RPE cells produce VEGF [32], which is preferentially secreted from the basal side towards the choroid. [25] On CECs, VEGFR2 is mainly expressed on the side of the choroid facing the RPE, suggesting that the survival of CECs depends on RPE-mediated signaling [25].

PEDF is a glycoprotein in the serpin family that has anti-angiogenic and neuro-protective properties [33, 34] and is secreted by the RPE [35]. It supports the morphogenesis and preserves the survival of photoreceptors, [36–38] and it maintains the quiescence of choroidal vessels [39]. Gao *et al*. have proposed that the expression of VEGF and PEDF maintains a delicate ratio and that this ratio is disrupted in CNV [40].

Multiple studies show that angiogenesis in many models is tightly regulated by epigenetic factors [41–43]. Epigenetics is defined as heritable changes in the chromatin structure leading to the regulation of gene expression, such as histone acetylation [44]. Histone deacetylase inhibitors (HDACi) have been shown in several cancer cell lines to elicit an anti-angiogenic effect [41–43]. HDAC7 inhibition in EC was shown to alter its migration, a key step in angiogenesis [45]. Crosson *et al*. demonstrated that damage to the eye caused by ischemia, one of the possible causative factors in CNV, can be reversed by the administration of trichostatin A (TSA) in a rat ischemic model [46]. In a recent publication, Crosson’s group showed that HDAC2 is crucial for mediating ischemic retinal injury, and the knockdown of this HDAC isoform can alleviate retinal degeneration caused by ischemia. [47] While an HDACi has been shown to inhibit experimental CNV, [48] the detailed mechanism of this effect has yet to be elucidated. In the current study, we attempted to determine first, whether the inhibition of histone deacetylases can regulate the activation of transdifferentiation of RPE cells. Second, we examined the effect of HDAC inhibition on the expression of angiogenic genes by RPE cells and angiogenesis *in vitro*. Third, we investigated how TSA modulated laser-induced CNV *in vivo*.

## Materials and Methods

### Cell culture

Human RPE cells were isolated in our laboratory from fetal human eyes of 18–20 weeks’ gestation (Advanced Bioscience Resources, Inc, Alameda, CA) [49]. The cells were cultured in Dulbecco’s modified Eagle’s medium (DMEM, Fisher Scientific, Pittsburgh, PA) with 2 mM L-glutamine, 100 U/mL penicillin, 100 μg/mL streptomycin (Sigma-Aldrich, St. Louis, MO), and 10% heat-inactivated fetal bovine serum (FBS; Irvine Scientific, Santa Ana, CA). The culture method we used is a standard practice in our laboratory and regularly yields >95% cytokeratin-positive RPE cells. Cells used were from passages 2 to 4. Cell treatments were performed on chamber slides, 6-well plates, or 96-well plates (BD Falcon, San Jose, CA) and were initiated 24 h after cell plating or subculture. CECs were isolated from bovine eyes using magnetic beads bound to the specific endothelial marker *Lycopersicon esculentum* (Sigma-Aldrich), as previously described. [50] Human umbilical vein endothelial cells (HUVECs) were purchased from ATCC (Manassas, VA). Both BCECs and HUVECs were cultured in endothelial growth medium (EGM Bullet Kit, #CC-3124, Lonza, Switzerland), and both cell types were used from passages 2 to 8.

### Cell Cycle and Cell Viability Analysis

For cell cycle analysis, RPE cells incubated in serum-free DMEM for 24 h and treated in serum-free DMEM with 0, 0.1, 0.5 or 1 μM TSA for 24 h were harvested, fixed in 1 mL of ice-cold 70% ethanol at room temperature for 10 min, and washed twice with ice-cold phosphate-buffered saline (PBS, pH 7.4). Each sample of 1×10^6^ cells was pelleted and re-suspended in 1mL of 10 μg/mL propidium iodide (Life Technologies, Grand Island, NY), incubated at 37°C for 30 min, and analyzed using an EPICS XL-MCL flow cytometer (Beckman Coulter, Irvine, CA). Samples of 5×10^3^ cells were used for analysis. The experiment was repeated three times. For cell viability analysis, RPE cells (5 × 10^4^) were seeded in 96-well plates in DMEM with 10% FBS. After overnight incubation to allow for cell attachment, the medium was replaced with serum-free DMEM, and the cells were treated with 0, 0.05, 0.1, 0.3, 0.5, 0.7 or 1 μM TSA (Sigma-Aldrich) in 90 μL of serum-free DMEM per well for 24 h. At the end of TSA treatment, 10 μL of PrestoBlue reagent (Life Technologies) was added per well. After a 6-hour incubation absorbance was read at 570 nm, using a reference wavelength of 600 nm for normalization, with a multi-well plate reader (Benchmark Plus, Bio-Rad, Tokyo, Japan). The experiment was repeated three times.

### Attachment Assay

Attachment was measured with a modified MTT [(3-(4,5-dimethylthiazol-2-yl)-2,5-diphenyltetrazolium bromide)] assay using 96-well plates coated with fibronectin (Life Technologies) (2 μg/cm^2^). After treatment with TSA (0.05, 0.1, 0.3, 0.5 or 0.7 μM) for 24 hours, RPE cells were trypsinized and re-suspended in DMEM with 0.4% FBS. 100 μL of cell suspension (10^4^ cells) were added to each well and allowed to attach for 5, 10, 15 or 30 min. The cells were washed gently with PBS twice, and 150 μL of fresh DMEM with 10% serum was added to each well with 20 μL of MTT (5 mg/mL; Sigma-Aldrich). After 4 hours of incubation at 37°C, the supernatants were decanted, the formazan precipitates were solubilized by the addition of 150 μL of 100% DMSO, and the plate was mixed on a plate shaker for 10 minutes. Absorbance at 550 nm was determined on a multiwell plate reader. The number of attached cells was proportional to the absorbance of MTT at 550 nm.

### Migration Assay

Migration was measured using a modified Boyden chamber assay, as previously described [51]. Briefly, 5 × 10^4^ RPE cells that were treated in serum-free DMEM with 0, 0.05, 0.1, 0.3, 0.5 or 0.7 μM TSA for 24 h were seeded in the upper part of a Boyden chamber together with the corresponding concentrations of TSA in 24-well plates. Inserts were coated with fibronectin (2 μg/cm^2^). The lower chamber was filled with 0.4% FBS-DMEM containing 10 ng/mL of recombinant PDGF-BB (R&D Systems Inc., Minneapolis, MN). After 5 hours of incubation, the inserts were washed three times with PBS, fixed with cold methanol (4°C) for 10 minutes, and counterstained with hematoxylin for 20 minutes. The number of migrated cells was counted by phase-contrast microscopy (×320). Four randomly chosen fields were counted per insert.

### Real-time polymerase chain reaction

RPE cells were pre-exposed to 0–0.5 μM TSA for 14 h. The cells were then treated with 150 μM cobalt chloride (CoCl_2_) (Sigma-Aldrich, St. Louis, MO), with or without TSA, for 6 h. Additional RPE cells were exposed to TSA only for 20 h. HUVECs were treated with 0–0.7 μM TSA for 48 h. Total RNA was extracted from the cells with TriZol (Life Technologies), and reverse transcription was performed with 1 μg of total RNA, using the ImProm-II Reverse Transcription System according to the manufacturer’s protocol (Promega, Madison, WI). Real-time PCR was performed in duplicate with a kit used according to the manufacturer’s recommendation (Roche Diagnostics, Indianapolis, IN). The primer sequences used are listed in Table 1. The quantity of mRNA was calculated by normalizing the threshold cycle values of VEGF, PEDF or VEGFR2 to the threshold cycle value of the housekeeping gene RPL13A of the same RNA sample, according to the published formula [52].

**Table 1:** Primer sequences used in real-time PCR.

Gene	Forward Primer	Reverse Primer
VEGF	5’CTACCTCCACCATGCCAAGTG 3’	5’ TGCGCTGATAGACATCCATGA 3
PEDF	5’CGACCAACGTGCTCCTGTCT 3’	5’ GATGTCTGGGCTGCTGATCA 3’
VEGFR2	5’ACTGCAGTGATTGCCATGTTCT 3’	5’ CCTTCATTGGCCCGCTTAA 3’
HIF-1α	5’CAGCAACTTGAGGAAGTACC 3’	5’ CAGGGTCAGCACTACTTCG 3’

### Western blot analysis

Confluent human fetal RPE cells grown in 6-well plates were starved for 24 hours in serum-free DMEM, and then treated with 0, 0.05, 0.1, 0.3, 0.5 or 0.7 μM TSA only for 24 h, or with 0, 0.05, 0.1, 0.3, or 0.5 μM TSA for 18 h, and then co-treated with 150 μM CoCl_2_ (Sigma-Aldrich) for 6 h, or pre-treated with 0, 0.1, 0.3, 0.5 or 0.7 μM TSA in DMEM with 0.1% FBS for 1h, and then co-treated with 20 ng/mL of human recombinant TGF-β1 (R&D Systems Inc.) for 72 hr. Bovine CECs (BCECs) or HUVECs grown in 6-well plates were starved for 4 h in endothelium basal medium (EBM, Lonza, Basel, Switzerland) with 1% FBS, and then treated with 0, 0.05, 0.1, 0.3, 0.5 or 0.7 μM TSA only for 24h or 48 h, or followed by stimulation with 20 ng/mL human recombinant VEGF (R&D Systems Inc Minneapolis, MN) for 10 min. Cells were harvested and lysed by RIPA buffer (Cell Signaling, Danvers, MA), and proteins were resolved on 4–15% Tris-HCl polyacrylamide gels (Bio-Rad) at 120 V. The proteins were transferred to polyvinylidene blotting membrane (Millipore, Bedford, MA). To assay HIF-1α, VEGF and PEDF, RPE cells that had been pre-exposed to TSA for 18 h were treated with CoCl_2_ for 6 h. To assay-pAkt, p-p42/44 and caspase 3, BCECs were treated with TSA for 24 h. To assay p-VEGFR2, HUVECs were treated with TSA for 48 h and then stimulated with 25 ng/mL human recombinant VEGF for 10 min. To assay VEGFR2, BCECs were treated with TSA for 24 h; HUVECs were treated with TSA for 48 h. The membranes were probed with the corresponding antibodies, as listed in Tables 2, 3 and 4.

**Table 2:** Antibodies used for RPE cells.

Primary Antibody	Primary dilution	Company	Catalog Number
Rabbit anti-VEGF	1:100	Santa Cruz Biotechnology, Inc.; Santa Cruz, CA	sc-152
Mouse anti-PEDF	1:100	R&D Systems Inc.; Minneapolis, MN	MAB1177
Mouse anti-HIF-1α	1:200	Novus Biologicals; Littleton, CO	NB100–105
Mouse anti-α-smooth muscle actin	1:1,000	Sigma-Aldrich	F3777

**Table 3:** Antibodies used for bovine choroidal endothelial cells.

Primary Antibody	Primary Dilution	Company	Catalog Number
Rabbit anti-p-p38	1:200	Cell Signaling Technology Inc., Danver, MA	9211
Rabbit anti-p-Akt	1:500	Cell Signaling Technology Inc., Danver, MA	9271
Rabbit anti-p-p42/44	1:500	Cell Signaling Technology Inc., Danver, MA	4377
Rabbit anti-caspase 3	1:100	Santa Cruz Biotechnology, Inc.; Santa Cruz, CA	sc-7148
Rabbit anti-p38	1:200	Cell Signaling Technology Inc., Danver, MA	9212
Rabbit anti-Akt	1:500	Cell Signaling Technology Inc., Danver, MA	9272
Mouse anti-p42/44	1:500	Cell Signaling Technology Inc., Danver, MA	9102

**Table 4:** Antibodies used for human umbilical vein endothelial cells.

Primary Antibody	Primary Dilution	Company	Catalog Number
Rabbit anti-p-VEGFR2	1:200	Santa Cruz Biotechnology, Inc.; Santa Cruz, CA	sc-101821
Rabbit anti-VEGFR2	1:200	Cell Signaling Technology Inc., Danver, MA	2479

Membranes were washed with TBS-Tween 20 (0.1%) (Bio-Rad) and then incubated with a horseradish peroxidase-conjugated secondary antibody (Vector Laboratories, Inc. Burlingame, CA) for 30 min at room temperature. To assay Akt, p42/44, or VEGFR2, the membranes were re-probed with the corresponding antibody, as listed in the tables. The subsequent procedures were performed as described above. Images were developed by adding enhanced chemiluminescence detection solution (Amersham Pharmacia Biotech, Cleveland, OH). Densitometry was performed using the Image J software (rsbweb.nih.gov/ij).

### TUNEL assay

BCECs cultured in chamber slides were starved for 4 h in endothelium basal medium with 1% FBS and then treated with 500 μM hydrogen peroxide (Sigma-Aldrich) for 4 h or with 0.7 μM TSA for 24 h. Terminal deoxynucleotidyl transferase dUTP nick end labeling (TUNEL) assay was performed using the *In Situ* Cell Death Detection Kit, TMR red (Roche Diagnostics, Mannheim, Germany), according to the manufacturer’s instructions. TUNEL-positive cells were counted under a fluorescence microscope (EVOS, Advanced Microscopy Group, Bothel, MA). The number of positive cells was presented as the percentage of dead cells.

### Tube formation assay

Confluent HUVECs were treated with 0, 0.3, 0.5, 0.7 or 1 μM TSA for 24 h. Two-dimensional tube formation was measured in Geltrex Reduced Growth Factor Basement Membrane Matrix gel (Life Technologies). The gel was placed in 96-well plates and incubated at 37°C for 30 min to reconstitute into basement membrane. Samples of 1 × 10^4^ HUVECs were seeded on each well and incubated with EBM + 1% FBS with TSA and human recombinant VEGF (25 ng/ml) for 2 h. Tube formation was documented by photography with a phase-contrast microscope. The experiment was repeated three times. The amount of tube formation was quantified using the Image J software.

### ChIP assay

ChIP assay was performed using the Imprint Chromatin Immunoprecipitation Kit (Sigma-Aldrich) according to the manufacturer’s instructions, with some modifications: 5×10^5^ human fetal RPE cells, untreated or treated in serum-free DMEM with 150 μM of CoCl_2_ alone for 6 h, or pre-treated with 0.3 μM of TSA for 18 h followed by co-treatment with 150 μM of CoCl_2_ for 6 h, or 5×10^5^ HUVECs, untreated or treated with 0.5 μM TSA in EBM with 1% FBS for 48 h, were used per assay, and the chromatin was first fixed with 1% formaldehyde for 10 min and then sonicated using the Branson Sonifier (Branson, Danbury, CT). The formaldehyde-fixed lysate was first pre-cleared with 5 μg of normal mouse IgG (Santa Cruz Biotechnology, Inc. Santa Cruz, CA) at 4°C for 45 min and then incubated together with Protein A agarose (Sigma-Aldrich) at 4°C for 45 min. The pre-cleared lysates were incubated with the assay wells coated with normal mouse IgG from Santa Cruz Biotechnology, anti-active RNA polymerase II from the kit, or anti-acetyl-histone H3 antibody (Millipore, Billerica, MA) overnight at 4°C. To release the chromatin, each reaction well was incubated with 40 μL of the DNA release solution with 1 μL of proteinase K at 65°C for 30 min and then with 40 μL of the reversing solution for 90 min. All chromatin samples and inputs were amplified by PCR, using the primer sequences listed in Table 5. The PCR reactions were run using ZymoTaq Premix (Zymo Research, Irvine, CA) in the MyCycler Thermocycler (Bio-Rad). Reaction conditions were as follows: 10 min at 95°C, followed by 35 cycles of 30 sec at 95°C, 30 sec at 56°C, 1 min at 72°C, and then 1 cycle of 7 min at 72°C (PEDF); 10 min at 95°C, followed by 35 cycles of 30 sec at 95°C, 30 sec at 54°C, 1 min at 72°C, and then 1 cycle of 7 min at 72°C (VEGF); 10 min at 95°C, followed by 40 cycles of 30 sec at 95°C, 30 sec at 56°C, 1 min at 72°C, and then 1 cycle of 7 min at 72°C (VEGFR2). The assay was performed three times. The PCR results were quantified with the Image J software.

**Table 5:** Primer sequences used in ChIP assay.

Gene	Forward Primer	Reverse Primer
PEDF	5’GAAGAGGAAGGTGTGCAAATG 3’	5’CCCAGCCTAGTCCCTCTAA 3’
VEGF	5’GCTTCACTGAGCGTCCGCA 3’	5’AATATCAAATTCCAGCACCGAGCGCC 3’
VEGFR2	5’ CGCGCTCTAGAGTTTCGGCAC 3’	5’ AGCGACCACACATTGACCGC 3’

### Laser-induced CNV and TSA injection

All procedures were performed in compliance with the Keck School of Medicine Institutional Animal Care and Use Committee approved protocols and the ARVO Statement for the Use of Animals in Ophthalmic and Vision Research (IACUC Protocol # 11710). Twenty wild type C57Bl/6 male mice, aged 6–8 weeks, were purchased from the National Cancer Institute (Frederick, MD) and kept, with 5 mice per cage, in shoebox cages with solid flooring that is covered with bedding material, in the vivaria at the Doheny Eye Institute. The mice were fed the standard laboratory chow in an air-conditioned room equipped with a 12-h light/12-h dark cycle. The mice were kept for 2 days before starting the experiment, and then randomly assigned to the control and experimental groups. Two groups of five mice each were included in two control groups that received intraperitoneal (IP) injections of PBS, one group for seven days of observation and the other for 14 days of observation. Two groups of five mice each were included in two experimental groups that received IP injections of TSA, one group for seven days of observation and the other for 14 days of observation. For all surgical procedures, the mice were anesthetized with an IP injection of 96 mg/kg of ketamine and 4.8 mg/kg of xylazine, and their pupils were dilated with topical 1% tropicamide (Alcon, Fort Worth, TX). Diode laser photocoagulation (75-μm spot size, 0.1-sec duration, 110 mW) was performed on both eyes of each mouse on day 0. Three laser photocoagulation burns were delivered to the retina lateral to the optic disc, through a slit lamp, with a coverslip used as a contact lens. Only lesions in which a subretinal bubble developed were used for experiments. Based on the fact that three lesions were induced per eye, and taken into considerations the variability in outcome, five mice per group are necessary to attain statistical significance. The TSA used in this study was obtained from Selleck Chemicals (Houston, TX). TSA was delivered to the mice by IP administration at 20 mg/kg per injection. Injections were made shortly after laser photocoagulation and every 48 h after laser treatment at about 1pm for 7 or 14 days, with the PBS control group injected first followed by the TSA group, and the mice were also checked for signs of discomfort. Control mice were injected with 0.5 mL of sterile PBS.

### Fluorescein angiography and histological analysis

The effect of TSA treatment on the development of CNV was evaluated on days 7 and 14 by semiquantitative assessment of late-phase fluorescein angiograms using the Kowa Genesis 35 mm fundus camera, captured 3 min after IP injection of 0.1 mL of 2.5% fluorescein sodium (Akorn, Decatur, IL), as previously described [53]. Ten animals, 20 eyes, and 34 lesions were examined in the control group, and 10 animals, 20 eyes, and 35 lesions were examined in the TSA group. Leakage was defined as the presence of a hyperfluorescent lesion that increased in size with time in the late-phase angiogram. Angiography was graded in a masked fashion by two examiners using reference angiograms. Angiograms were graded as follows: 0, no leakage; 1, slight leakage; 2, moderate leakage; and 3, prominent leakage [52]. While under anesthesia, cervical dislocation was performed and the animals were checked for lack of breathing, lack of a heartbeat and the body becoming cold. The right eye from each mouse was used for histologic analysis while the left eye was used for CNV volume analysis (see next section). For histopathologic analysis, enucleated eyes were snap frozen. Sections (8 μm) from the center of the lesion were stained with hematoxylin and eosin (H&E), to assess the histology of the retina with TSA treatment, laser lesions, and subsequent CNV development. Measurement of CNV lesion area in the H&E-stained sections was carried out with the ImageScope software (Aperio, Vista, CA). Eight animals, eight eyes and eight lesions were examined in the control group, and nine animals, nine eyes and nine lesions were examined in the TSA group.

### CNV volume analysis

Eyes were enucleated on days 7 and 14 after fluorescein angiography and euthanasia and fixed with 10% formalin overnight at 4°C. Eye cups were obtained by removing the anterior segments and neurosensory retina and washed three times in PBS. The remaining eye cups containing the RPE—choroid—sclera complex were incubated with blocking buffer (PBS containing 1% BSA and 0.5% Triton X-100) for 1 h at room temperature. The eye cups were stained with 10 μg/mL of FITC-isolectin B4 and then visualized using the 20× objective of a scanning confocal microscope (model LSM510; Carl Zeiss Meditec, Inc, Thornwood, NY). Fluorescence volume measurements were made by creating image stacks of optical slices within lesions. The image stacks were generated in the Z-plane, with the confocal microscope set to excite at 488 nm and to detect at 505 to 530 nm. Images were further processed using the microscope’s system software (LSM; Carl Zeiss Meditec, Inc.), by closely circumscribing and digitally extracting the fluorescent lesion areas throughout the entire image stack. The extracted lesion was processed through the topography software to generate a digital topographic image representation of the lesion and an image volume. The topographic analysis program determines and displays the objects’ surface contours by detecting fluorescent signal from the top of the image stack and then measures everything under the surface to yield a final volume (square micrometers ± SD) that reflects the CNV fluorescence volume [54]. Ten animals, 10 eyes and 20 lesions were examined in the control group, and 10 animals, 10 eyes and 28 lesions were examined in the TSA group.

### Immunohistochemistry

Antibodies are listed in Table 6. Cryostat sections (8 μm) of snap frozen mouse eyes were prepared from animals treated with PBS or TSA from day 7 of the CNV model. The slides were fixed with methanol for 10 min, and then rinsed in PBS for 5 min. After fixation, the slides were incubated with 0.5% hydrogen peroxide for 5 min, followed by 5% normal goat serum for 15 min. Next, sections were incubated with primary antibodies overnight at 4°C. Binding of the primary antibody was visualized first by incubating with biotinylated anti-rabbit or mouse IgG antibody (Vector Laboratories) for 30 min, and then with horseradish peroxidase-conjugated streptavidin (Life Technologies) for 30 min. Sections were counter-stained with hematoxylin for 2 min, mounted in aqueous mounting medium (Vector Laboratories), and examined and quantified using the Aperio ScanScope CS, 20× lens with doubler for 40×, and the ImageScope software (Aperio).

**Table 6:** Antibodies used for immunohistochemistry.

Primary Antibody	Primary Dilution	Company	Catalog Number
Rabbit anti-VEGFR2	1:200	Cell Signaling Technology Inc., Danver, MA	2479
Mouse anti-VEGF	1:500	Abcam; Cambridge, MA	ab1316

### Evaluation of TSA *in vivo* Toxicity

The toxicity of TSA on normal choroidal vasculature was evaluated in wild type male 6–8 week old C57Bl/6 mice after 14 days of systemic therapy. Three mice were assigned each to the TSA and PBS control groups, and they were intraperitoneally injected with 20 mg/kg TSA or the same volume of PBS in the same regiment as the mice in the CNV model. The animals were euthanized on day 14, and eye cups were isolated from enucleated eyes as described in “CNV Volume Analysis”, and then the RPE layer was gently washed off. Afterwards, the choroid-sclera complex was fixed in 4% paraformaldehyde at room temperature for 20 min, and then washed with PBS at room temperature for 30 min. TUNEL assay was performed using the *In Situ* Cell Death Detection Kit, TMR red (Roche), according to the manufacturer’s instructions, except that the permeabilization time was increased to 5 min. Positive controls consisted of flatmounts and sections treated with 2000 U/ml of rDNase 1 (Sigma). The flatmounts were incubated with 10 μg/mL of FITC-isolectin B4, mounted with DAPI and then visualized using the 10× and 40× objectives of the PerkinElmer Ultraviewer Spinning Disk Confocal Microscope (PerkinElmer, Waltham, MA). Three animals and six eyes were examined in the control groups, and three animals and six eyes were examined in the TSA group. TSA toxicity was also evaluated in the cryosections from the laser CNV mouse model by TUNEL assay using the *In Situ* Cell Death Detection Kit, TMR red, according to the manufacturer’s instructions. The cryosections were then incubated with 10 μg/mL of FITC-isolectin B4, mounted with DAPI and then visualized with Spinning Disk Confocal Microscope PerkinElmer Ultraviewer. Three animals and three eyes were each examined in each of the PBS and TSA groups.

### Statistical analysis

All *in vitro* experiments were repeated at least three times, the number of animals, number of eyes and number of lesions examined in the *in vivo* experiments were listed above, and were all analyzed by the Student’s *t* test. Analysis of variance (one-way ANOVA) was performed to test the statistical significance for cell cycle analysis, PrestoBlue assay, attachment assay, all Western blots, all real-time PCR, migration assay, TUNEL assay, and tube formation assay.

## Results

### TSA arrests RPE cell cycle progression

**Fig 1:**
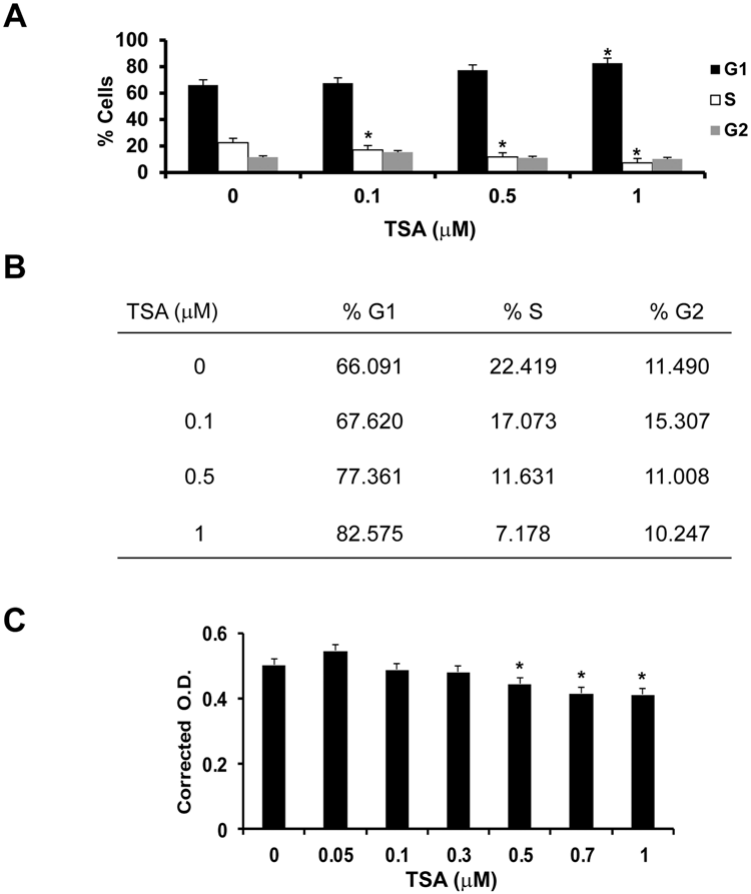
TSA induces RPE cell cycle arrest by inhibiting cell proliferation. Cell cycle analysis of RPE cells treated with 0–1 μM TSA for 24 h, fixed with ice-cold 70% ethanol and stained with propidium iodide by an EPICS XL-MCL flow cytometer. (A) With increasing doses of TSA, significantly more RPE cells were in the G1 phase and fewer in the S phase than untreated cells. (*: t test p<0.01) (B) Percentage of cells in each phase of the cell cycle after TSA treatment. (C) PrestoBlue assay was performed on RPE cells treated with 0, 0.05, 0.1, 0.3, 0.5, 0.7 or 1 μM TSA to determine the toxicity of TSA on RPE cells. The lowest number of viable RPE cells was seen when cells were treated with 1 μM TSA for 24 h (82.0% viable cells). (*: t test p< 0.0001).

RPE cells were treated in serum-free DMEM with 0–1 μM TSA for 24 h. TSA caused a dose-dependent increase in the percentage of RPE cells in the G1 phase and a reduction of cells in the S phase (Fig. 1A). At 1 μM TSA, the percentage of RPE cells in G1 phase was at its highest (82.6%, t test: p<0.01; ANOVA: p<0.008) and that in S phase (7.2%, t test: p<0.01; ANOVA: p<0.004) was at the lowest level (Fig. 1B). This suggests that TSA suppresses RPE cell proliferation by inhibiting its cell cycle progression at the G1 phase. To determine whether the inhibition of cell cycle progression was associated with decreased cell number, we evaluated viable cell number by PrestoBlue assay. RPE cells were treated in serum-free DMEM with 0–1 μM TSA for 24 h, and then incubated with the PrestoBlue reagent for 6 h. The effect of TSA was small but statistically significant, which reduced the viable RPE cell number to 82.4% at 1 μM of TSA (Fig. 1C, t test: p<0.0001; ANOVA: p<0.0001).

### TSA promotes RPE cell attachment to fibronectin

**Fig 2:**
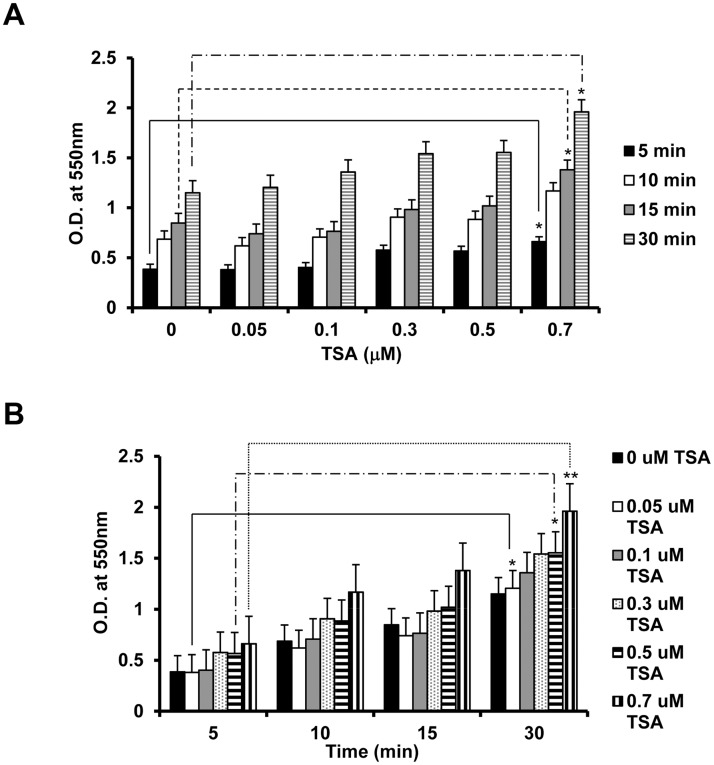
TSA promotes RPE cell attachment to fibronectin. RPE cells treated with 0–0.7 μM TSA for 24 h were allowed to attach to 96-well plates coated with fibronectin for 5, 10, 15 or 30 min. The attachment of RPE cells to fibronectin was evaluated by a modified MTT assay. Absorbance was read at 550 nm. TSA-treated RPE cells showed a (A) dose- and (B) time-dependent increase in attachment to fibronectin. (*: t test p<0.05; **: t test p<0.01).

RPE cell attachment to fibronectin was examined. RPE cells were treated in serum-free DMEM with 0–0.7 μM TSA for 24 h, and then incubated in DMEM with 0.4% FBS and the corresponding concentrations of TSA in 96-well plates for 5–30 min. At 15 min, cells treated with 0.5 μM and 0.7 μM of TSA, and at 30 min, cells treated with 0.3 μM and 0.7 μM of TSA, attachment to fibronectin was significantly increased. Maximal enhancement of attachment (348%, compared to untreated cells allowed to attach to fibronectin for 5 min) was observed at 0.7 μM of TSA at 30 min. (Fig. 2; t test: p<0.05; ANOVA: 15 min: p<0.03, 5 min, 10 min and 30 min: p>0.05)

### TSA inhibits RPE cell migration induced by PDGF

**Fig 3:**
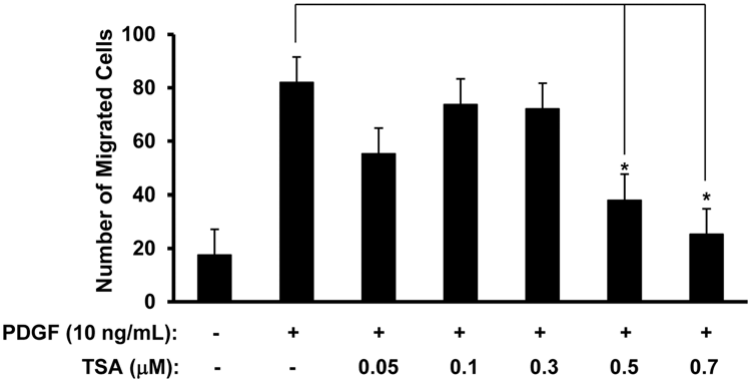
TSA inhibits PDGF-induced RPE cell migration. RPE cells treated with 0–0.7 μM TSA for 24 h were placed on the top of a fibronectin coated-Boyden chamber, with 10 ng/mL of PDGF and DMEM with 0.4% FBS added to the bottom chamber and incubated for 5 h. Four randomly chosen fields were counted per inserts, and the results presented are averages of the four fields from each insert from three independent experiments. TSA inhibits RPE cell migration at 0.5 and 0.7 μM. (*: t test p<0.05).

PDGF stimulated migration of RPE cells across the Boyden chamber insert. RPE cells were treated in serum-free DMEM with 0–0.7 μM TSA for 24 h, and then incubated in DMEM with 0.4% FBS with the corresponding concentrations of TSA in the inserts placed in 24-well plates for 5 h, with the lower chambers filled with DMEM containing 0.4% FBS and 20 ng/mL of PDGF-BB as the chemoattractant. Exposure to TSA at 0.5 μM and 0.7 μM significantly inhibited the PDGF-induced RPE cell migration in a dose-dependent manner. Maximal inhibition was observed at 0.7 μM TSA (69%, compared to cells exposed to PDGF alone). (Fig. 3; t test: p<0.05; ANOVA: p<0.02)

#### TSA inhibits the expression of TGF-β-induced α-smooth muscle actin

**Fig 4:**
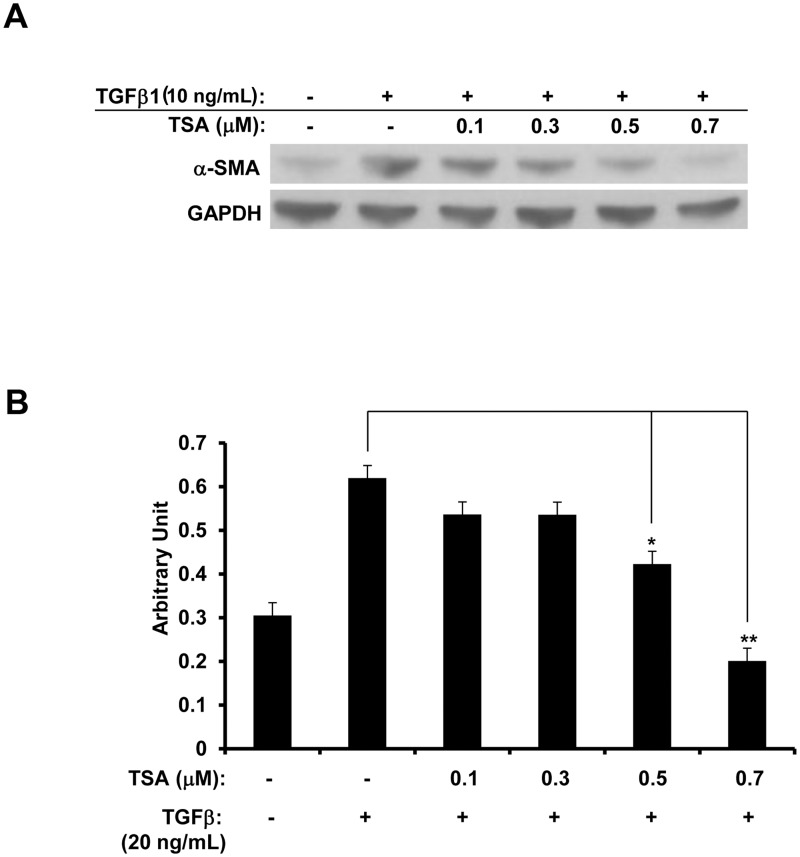
TSA suppresses α-SMA induced by TGF-β. RPE cells were treated with 20 ng/mL of TGF-β only, or co-treated with 0–0.7 μM TSA and 20 ng/mL of TGF-β for 72 h. (A) The expression of α-SMA is induced by TGF-β treatment by 2-fold, and this up-regulation was reduced by TSA in a dose-dependent manner. (B) Densitometry data for Western blot result of α-SMA. (*: t test p<0.05; **: t test p<0.01).

α-smooth muscle actin (α-SMA) is a marker for epithelial-mesenchymal transdifferentiation [14]. RPE cells were pre-treated in DMEM containing 0.1% FBS with 0–0.7 μM TSA for 1 h, and then co-treated with 20 ng/mL of TGF-β1 for 72 h. TGF-β up-regulated α-SMA expression, yet at 0.5 μM and 0.7 μM, TSA inhibited the TGF-β-induced expression of α-SMA, (Fig. 4; t test: p<0.05; ANOVA: p<0.01) suggesting that TSA can inhibit TGF-β-induced EMT in RPE cells.

### TSA down-regulates HIF-1α and VEGF and up-regulates PEDF

**Fig 5:**
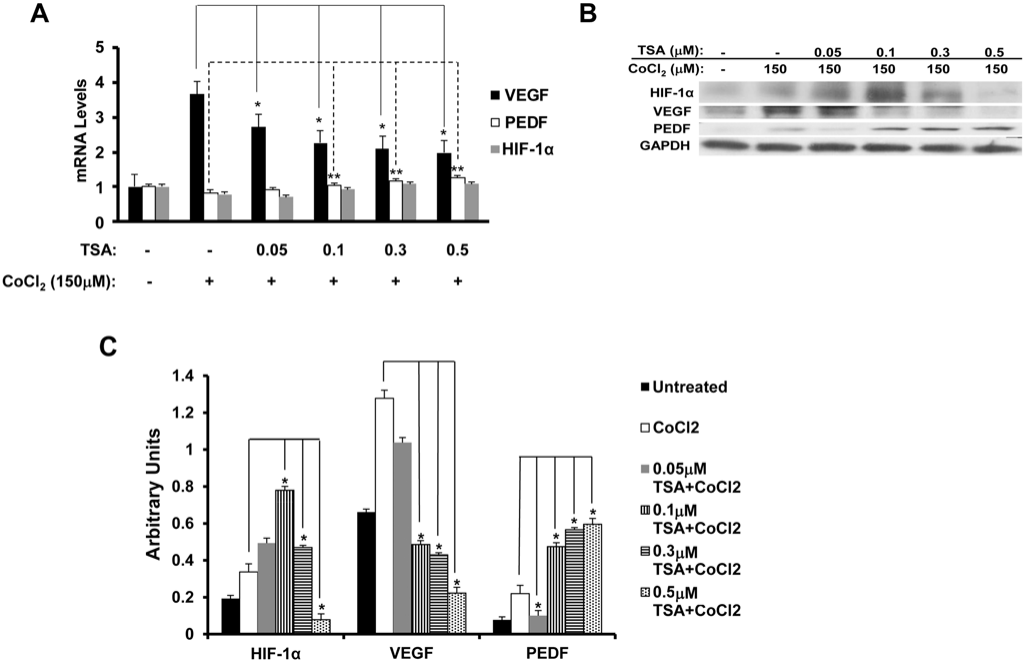
TSA reduces HIF-1α and VEGF expression and up-regulates the expression of PEDF. Real-time PCR (A) and Western blot assays (B) were performed on RPE cells treated with 0–0.5 μM TSA and 150 μM CoCl_2_. (A) Cells were treated with 0–0.5 μM TSA for 14 h and then co-treated with 150 μM CoCl_2_ for 6 h for the analysis of gene expression by real-time PCR. Changes in HIF-1α mRNA levels were not statistically significant. CoCl_2_ causes a fourfold enhancement of VEGF mRNA expression; but at 0.5 μM TSA, the mRNA level of VEGF reduces to less than half of that in cells treated with CoCl_2_ only. TSA induces a statistically significant increase in the mRNA level of PEDF. (B) Cells were treated with 0–0.5 μM TSA for 18 h and then co-treated with 150 μM CoCl_2_ for 6 h for Western blot analysis. TSA reduces the CoCl_2_-induced HIF-1α and VEGF protein levels by 4.3-fold and 5.7-fold, respectively, and up-regulates PEDF protein level by threefold. (C) Densitometry data for Western blot of HIF-1α, VEGF and PEDF. (*: t test p<0.05; **: t test p<0.01).

One possible mechanism of the inhibition of angiogenesis by TSA is through the down-regulation of HIF-1α and VEGF [47] and up-regulation of PEDF [55]. To stimulate VEGF expression we treated human RPE cells with a hypoxia-mimicking agent, CoCl_2_, for 6 h in serum-free DMEM after pre-treatment with 0–0.5 μM TSA for 14 h for real-time PCR, and 18 h of TSA pre-treatment for Western blot. As shown by real-time PCR, the levels of HIF-1α mRNA were not significantly changed; but at all concentrations of TSA used, VEGF mRNA level was down-regulated even in the presence of CoCl_2_ (Fig. 5A; t test: *p<0.05, **p<0.01; ANOVA: VEGF: p<0.01, PEDF: p<0.01, HIF-1α: p>0.05). At the same time, the anti-angiogenic and neuro-protective PEDF mRNA level was up-regulated when RPE cells were exposed to 0.5 μM of TSA used (Fig. 5A). To examine the protein expression of HIF-1α, VEGF and PEDF, Western blot was performed on RPE cells that had received the same treatment. TSA reduced the levels of HIF-1α and VEGF protein and raised the expression of PEDF protein in the presence of CoCl_2_ (Fig. 5B and 5C; t test: p< 0.05; ANOVA: VEGF, PEDF and HIF-1α: p<0.05).

### TSA treatment modifies transcriptional activity on VEGF and PEDF promoters

**Fig 6:**
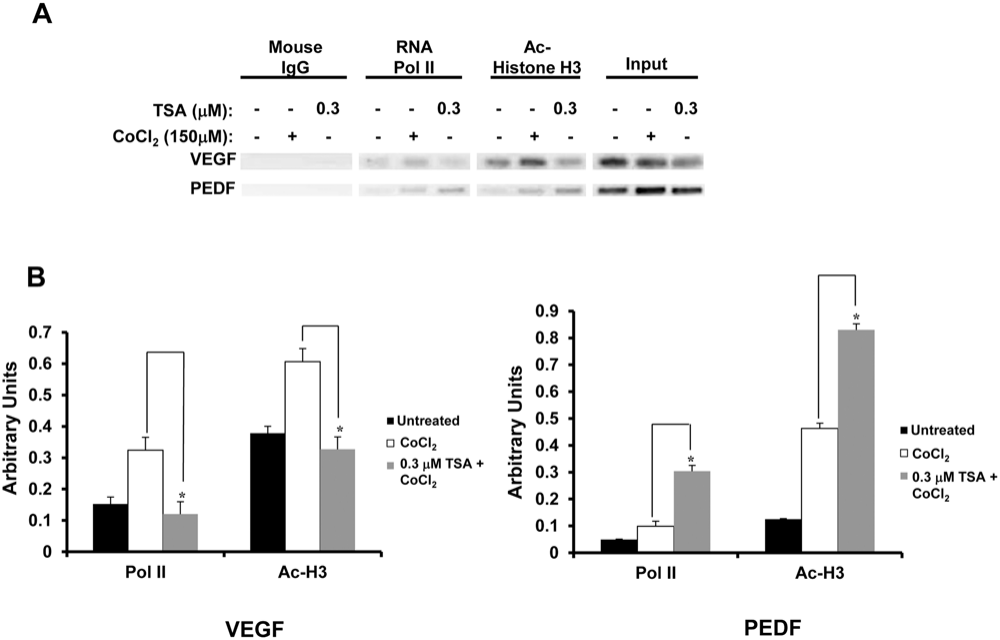
TSA regulates promoter activities of VEGF and PEDF. After RPE cells were harvested, chromatin fixed with 1% formaldehyde and fragmented by sonication was immunoprecipitated with mouse IgG, anti-RNA polymerase II or anti-acetyl-histone H3 antibodies. Released chromatin was then amplified by PCR using primers targeting VEGF and PEDF encompassing the region from 200 bp upstream of the transcription start site to 200 bp downstream of the transcription start site. Amplified chromatin was then run on a 1% agarose gel. (A) For VEGF, less promoter opening was found in TSA and CoCl_2_-treated cells than in CoCl_2_-only-treated cells and untreated cells. For PEDF, more promoter opening was found in TSA and CoCl_2_-treated cells than in CoCl_2_-only-treated cells and untreated cells. (B) Densitometry of ChIP assay result normalized by input levels. (*: t test p<0.05).

To examine whether the effect of TSA on the protein and mRNA levels of VEGF and PEDF were due to regulation at the promoters of these genes, ChIP assay was performed with lysed RPE cells. Chromatin from untreated RPE cells or RPE cells treated in serum-free DMEM with 150 μM of CoCl_2_ for 6 h, or pre-treated with 0.3 μM TSA for 18 h and then co-treated with 150 μM of CoCl_2_ for 6 h, was immunoprecipitated using antibodies targeting RNA polymerase II and acetyl-histone H3. The presence of these two proteins on the promoters indicates promoter opening at these genes and that these genes are actively being transcribed. CoCl_2_ treatment resulted in more chromatin pulled down by both antibodies for both VEGF and PEDF, indicating that transcription of these two genes was increased by CoCl_2_. There was a statistically significant decrease in promoter opening in TSA and CoCl_2_-treated cells, compared to CoCl_2_-only-treated and untreated cells, for VEGF, while there was a statistically significant increase in promoter opening in TSA and CoCl_2_-treated cells, compared to CoCl_2_-only-treated and untreated cells, for PEDF (Fig. 6; p<0.05), indicating VEGF and PEDF expression may be epigenetically regulated.

### TSA increases apoptosis in BCECs

**Fig 7:**
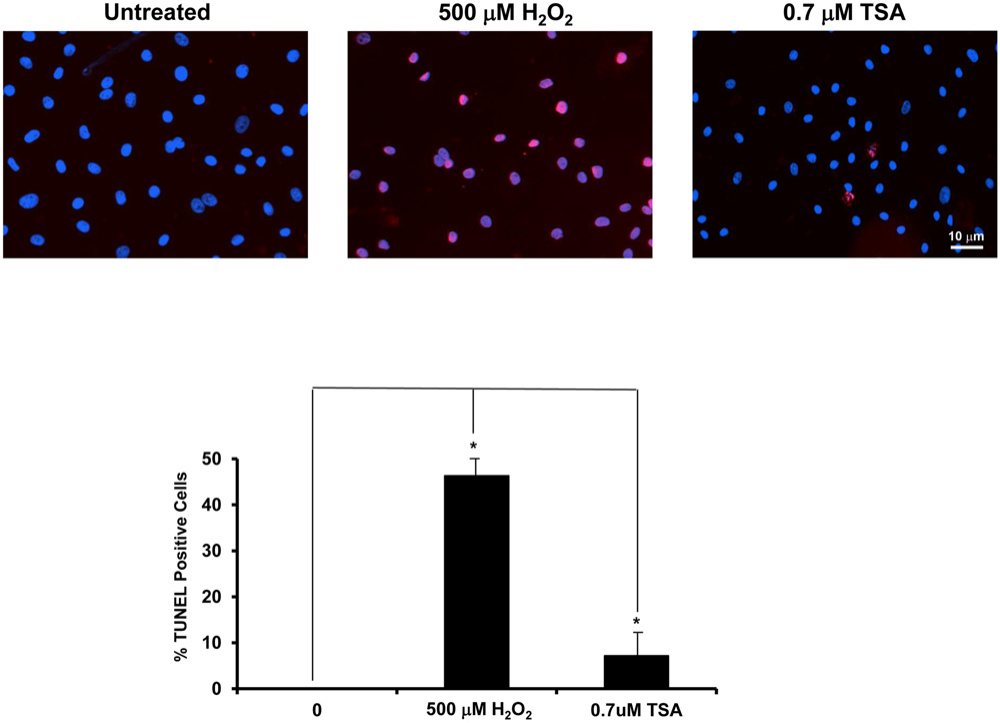
TSA increases apoptotic BCECs. TUNEL assay was performed on BCECs treated with 500 μM hydrogen peroxide for 4 h, or 0.7 μM TSA for 24 h. TSA moderately increases the number of TUNEL-positive BCECs. (*: t test p<0.0002) (bars = 10 μm).

To visualize the effect of TSA on BCEC apoptosis, we performed TUNEL assay on BCECs treated with 0.7 μM TSA in EBM with 1% FBS for 24 h, the highest level of TSA used in the experiments. BCECs treated with 500 μM hydrogen peroxide for 4 h were used as positive control. TSA-treated BCECs exhibited some TUNEL-positive cells (7.2%, p<0.0002, Fig. 7) as compared to untreated control, but much less than the oxidant treated positive control (46.3%, t test: p<0.00007; ANOVA: p<0.0001).

### TSA regulates BCEC cell signaling

**Fig 8:**
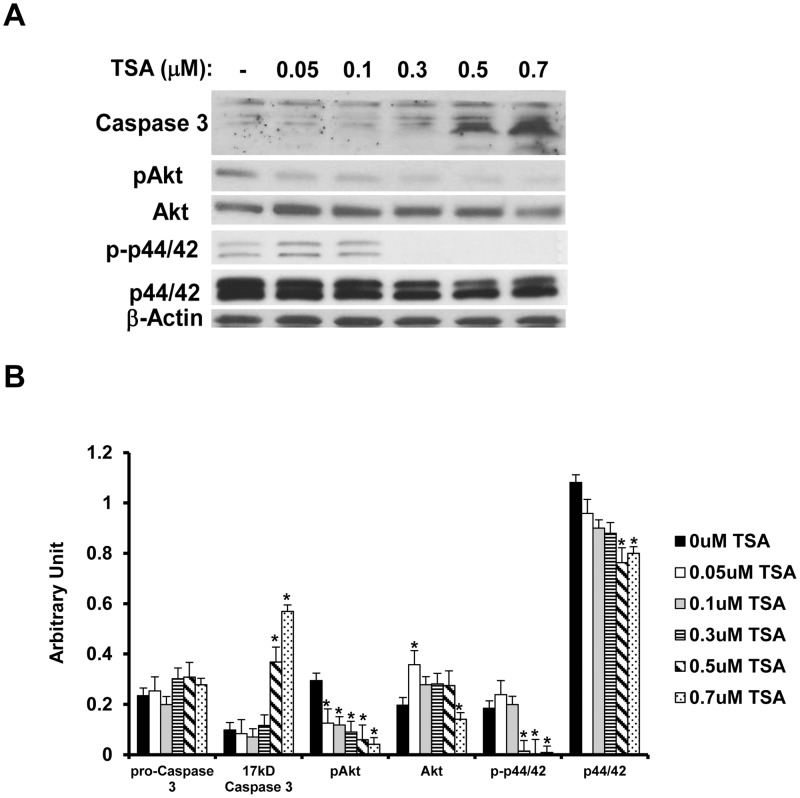
TSA activates caspase 3 but inhibits the activation of Akt and p42/44 in BCECs. (A) BCECs were treated with 0–0.7 μM TSA for 24 h. TSA activates caspase 3 at the concentrations of 0.5 and 0.7 μM, and blocks Akt phosphorylation in a dose-dependent manner. In untreated cells and cells treated with low concentrations of TSA (0.05–0.1 μM), p42/44 was activated, but the activation was completely obliterated at higher TSA concentrations (0.3–0.7 μM). (B) Densitometry data for Western blot results. (*: t test p<0.05).

To study how TSA inhibits new blood vessel formation, we investigated the effect of TSA on BCECs’ cell signaling events. BCECs were treated in EBM containing 1% FBS with 0–0.7 μM TSA for 24 h. TSA-treated BCECs exhibited an increase of activated caspase 3 levels but a down-regulation of phospho-Akt, while inhibiting the phosphorylation of p42/44 (Fig. 8; t test: p<0.05; ANOVA: pro-caspase: p>0.05, 17 kD caspase 3: p<0.0001, pAkt: p<0.0008, Akt: p<0.01, p-p42/44: p<0.001, p42/44: p<0.002); which suggests that TSA has an anti-proliferative and a moderate pro-apoptotic effect on BCECs.

### TSA inhibits tube formation in HUVECs

**Fig 9:**
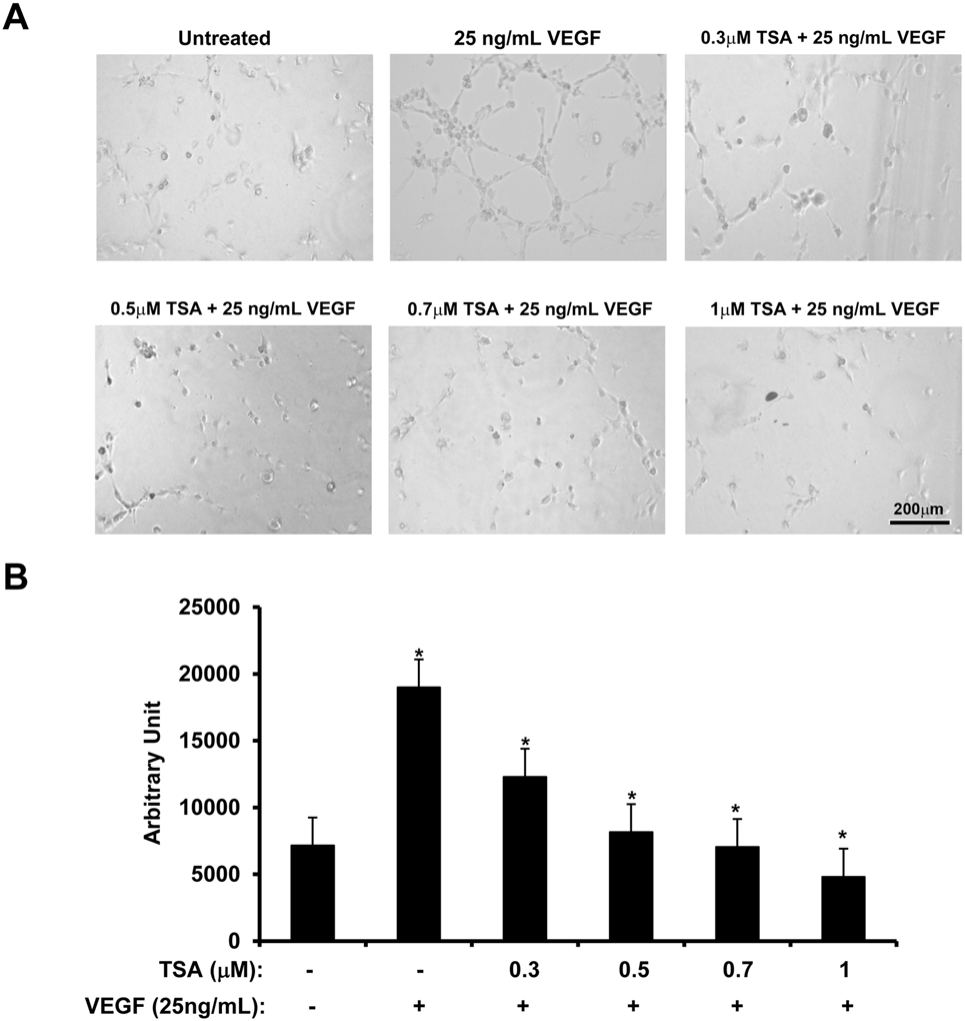
TSA impedes VEGF-induced tube formation in HUVECs. 1 × 10^4^ of HUVECs treated with 0–1 μM TSA for 24 h were transferred in 150 μL of endothelium basal medium + 1% FBS with or without 25 ng/mL of human recombinant VEGF and the corresponding concentrations of TSA then incubated on 50 μL of Geltrex Reduced Growth Factor Basement Membrane Matrix gel in 96-well plates for 2 h. (A) Phase-contrast microscopy documented that tube formation induced by VEGF was inhibited by TSA in a dose-dependent manner. (B) Quantification of the amount of tube formation under different treatment conditions. (*: t test p<0.05) (bar = 200 μm).

To assess the efficacy of TSA in inhibiting angiogenesis, untreated HUVECs and HUVECs treated with 0.3, 0.5, 0.7 or 1 μM TSA in EBM with 1% FBS for 24 h were tested for tube formation. Untreated HUVECs showed a limited amount of tube formation. In the presence of 25 ng/mL of VEGF, extensive tube formation occurred with prominent branching. Tube formation induced by VEGF was diminished in a dose-dependent manner with increasing doses of TSA. (Fig. 9A and 9B; t test: p<0.05; ANOVA: p<0.05)

### TSA down-regulates VEGFR2 in BCECs and HUVECs

**Fig 10:**
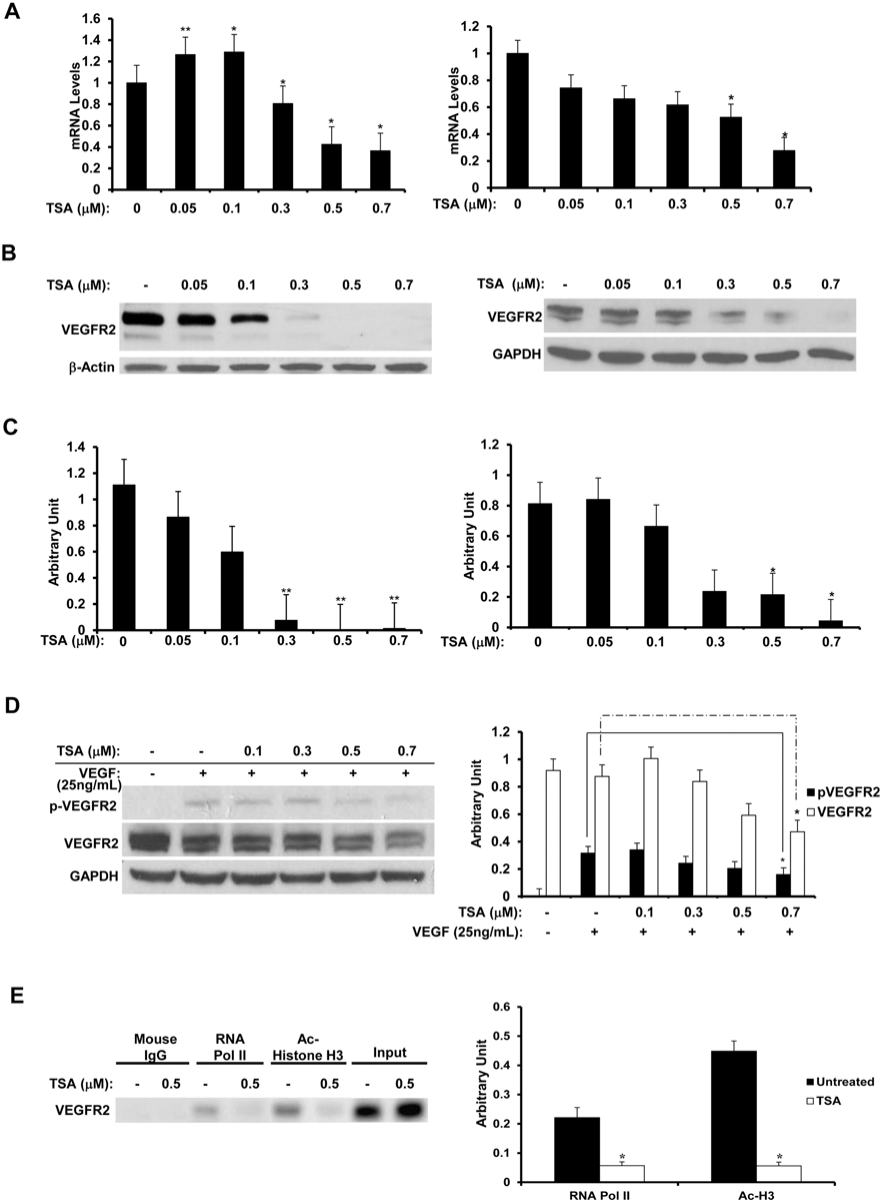
TSA inhibits VEGFR2 in BCECs and HUVECs. Real-time PCR (A) and Western blot (B) were performed on BCECs (left panel) and HUVECs (right panel) treated with 0–0.7 μM TSA for 24h or 48 h. (A-B) TSA exerts a dose-dependent reduction effect on the mRNA and protein levels of VEGFR2. (C) Densitometry data for Western blot result of VEGFR2 normalized by GAPDH levels for BCECs (left panel) and HUVECs (right panel). (*: t test p<0.05, **: t test p<0.01) (D) HUVECs were treated with 0–0.7 μM TSA for 48 h, and then stimulated with 25 ng/mL of human recombinant VEGF for 10 min, followed by Western blot analysis (left panel). VEGF significantly induces the phosphorylation of VEGFR2, but the phosphorylation was attenuated by TSA at 0.7 μM, concomitant with a down-regulation of VEGFR2 total protein level. Densitometry data (right panel) for Western blot result of VEGFR2 and phospho-VEGFR2 normalized by GAPDH levels. (*: t test p<0.05) (E) ChIP assay was performed as described in Fig. 3 on HUVECs treated with 0.5 μM TSA for 48 h. Released chromatin was then amplified by PCR using primers targeting VEGFR2 encompassing the region from 200 bp upstream of the transcription start site to 200 bp downstream of the transcription start site. Amplified chromatin was then run on a 1% agarose gel. Less promoter opening was found in TSA-treated cells than in untreated cells. Densitometry of ChIP assay result normalized by input levels. (*: t test p<0.05).

To study whether TSA can inhibit VEGFR2 expression, BCECs were treated in EBM containing 1% FBS with 0–0.7 μM TSA for 24 h, and the cell lysates were analyzed by real-time PCR and Western blot. TSA down-regulated VEGFR2 mRNA and protein expression in a dose-dependent manner; both isoforms were prominently down-regulated in the same decreasing pattern in Western blot. (Fig. 10A-C, left panels; t test: *: p<0.05, **: p<0.01; ANOVA: real-time PCR: p<0.01, Western blot: p<0.0001) Interestingly, the mature, higher molecular weight VEGFR2 was the dominant isoform expressed in BCECs. (Fig. 10B) However, the supply of BCECs was limited, and thus the effect of TSA on VEGFR2 was also examined in HUVECs. HUVECs were treated in EBM containing 1% FBS with 0–0.7 μM TSA for 48 h, and the cell lysates were analyzed by real-time PCR and Western blot. A similar dose-dependently reduced expression in VEGFR2 mRNA and protein levels was also observed in HUVECs. (Fig. 10A-C, right panels; t test: p<0.05; ANOVA; real-time PCR: p<0.01, Western blot: p<0.01)

To examine more in depth the effects of TSA on the signaling of VEGFR2, the phosphorylation of VEGFR2, indicating the activation of the receptor, was studied by treating HUVECs in EBM containing 1% FBS with 0–0.7 μM TSA for 48 h, and then stimulating them with 25 ng/mL of VEGF for 10 min. As expected, VEGF induced the phosphorylation of VEGFR2 (Fig. 10D); but at the highest dose of TSA used, the phosphorylation of VEGFR2 was reduced, while total VEGFR2 protein was also down-regulated. Densitometric analyses of phospho-VEGFR2 and total VEGFR2 levels normalized by GAPDH levels showed dose-dependent reductions by TSA (Fig. 10D; t test: p<0.05; ANOVA: p<0.01); whereas densitometric analysis of phospho-VEGFR2 levels normalized by total VEGFR2 protein levels displayed no statistically significant changes among the different TSA concentrations used (data not shown), demonstrating that the reduced levels of activated VEGFR2 was caused by decreased total VEGFR2 protein levels. Our result indicates that TSA attenuates VEGF-mediated pro-angiogenic signaling via VEGFR2 in ECs.

TSA treatment modifies transcriptional activity on VEGFR2 promoter in HUVECs ChIP assay was employed, as described above, to determine whether promoter modulation was involved in the down-regulation of VEGFR2 in HUVECs. HUVECs were untreated or treated in EBM containing 1% FBS with 0.5 μM TSA for 48 h. Compared to untreated cells, TSA treatment led to less promoter opening on the VEGFR2 gene (Fig. 10E; p<0.05), as the acetyl-histone H3 and RNA polymerase II antibodies pulled down less chromatin in TSA-treated cells, indicating that transcription activity at this promoter was reduced in TSA-treated HUVECs. This suggests that the regulation of mRNA and protein expression of VEGFR2 in HUVECs is likely regulated by epigenetics.

### TSA inhibits laser-induced CNV in mice

**Fig 11:**
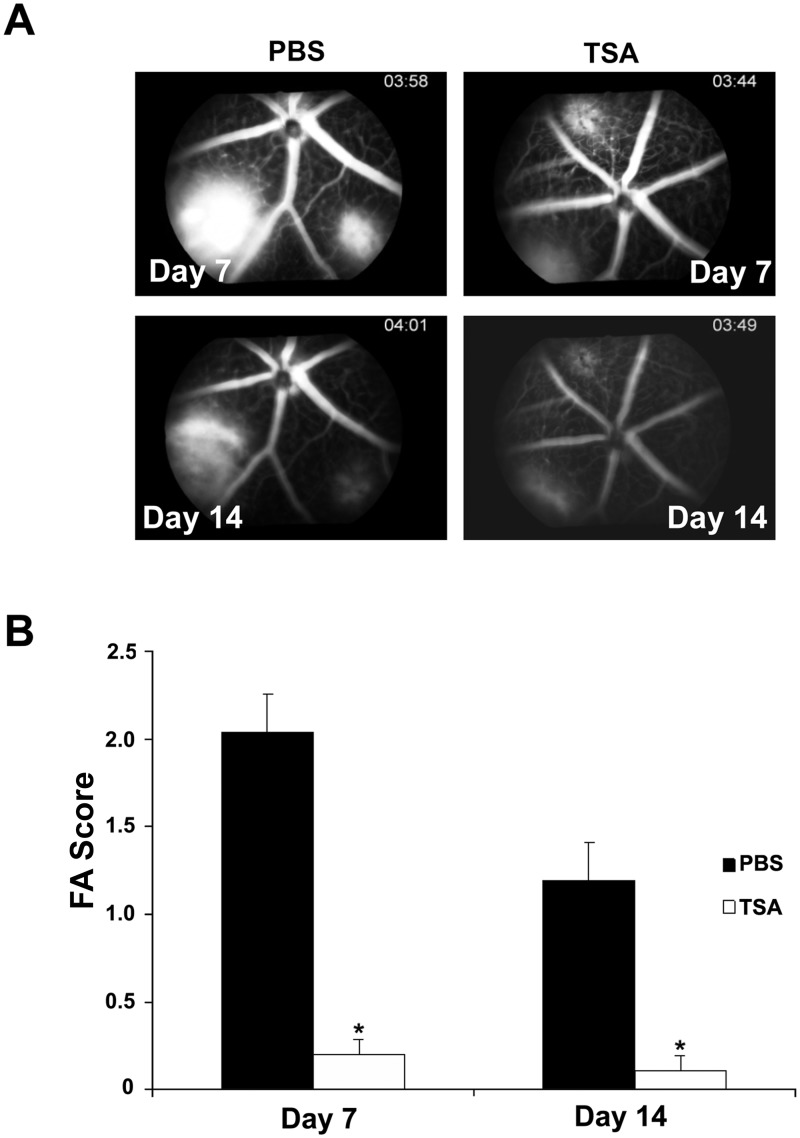
TSA reduces fluorescence leakage in laser-induced mouse CNV model. After laser photocoagulation on day 0, C57Bl/6 mice received intra-peritoneal injections of either PBS or TSA (20 mg/kg) every 48 h for 7 or 14 days. (A, B) Angiogram pictures were taken on days 7 and 14, 3 min after a 0.1 mL of 2.5% fluorescein sodium injection. For both days 7 and 14, TSA-injected mice showed significantly less fluorescence leakage than PBS-treated mice. (*: t test p<0.05; n = 10 mice/group).

**Fig 12:**
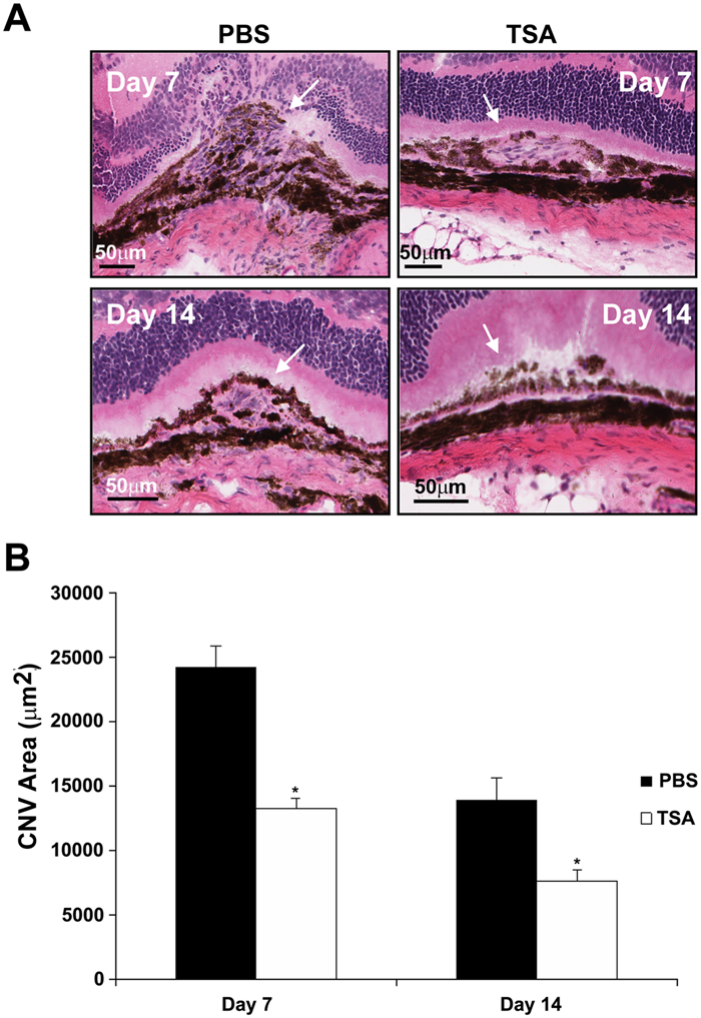
Histological analysis of mouse CNV lesions after TSA treatment. 8 μm sections were stained with hematoxylin and eosin to study the histology of CNV lesions induced by laser photocoagulation in mice treated with PBS or TSA every 48 h for 7 or 14 days. (A, B) On both days 7 and 14, the area of the CNV lesions was significantly smaller in the TSA-treated mice than in mice that received only PBS. White arrows denote the location of lesions. (*: t test p<0.05; n = 8 mice in PBS group, n = 9 mice in TSA group; bars = 50 μm)

**Fig 13:**
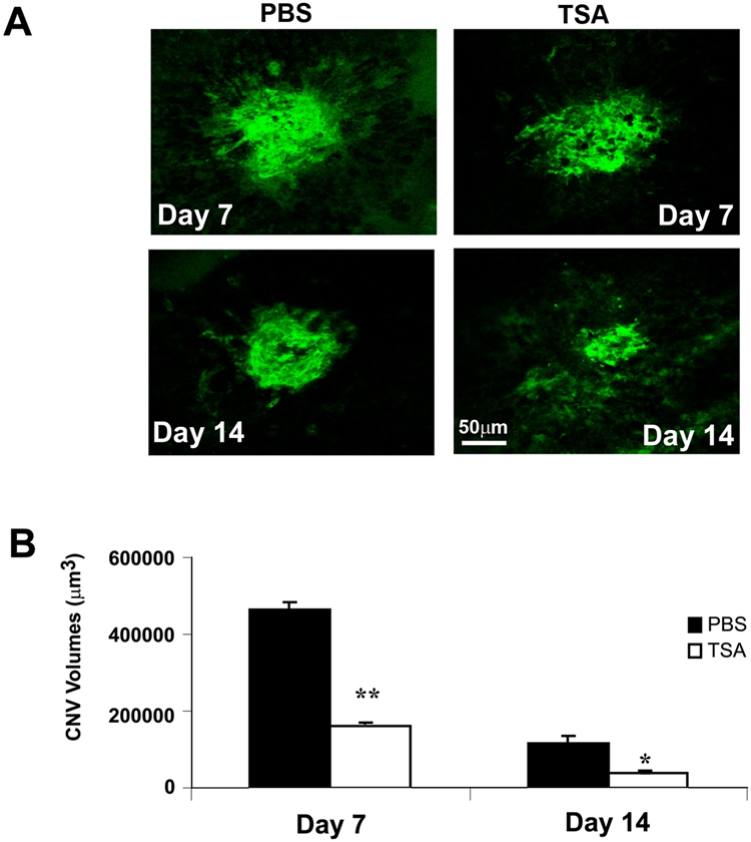
CNV volume measurements after TSA treatment. Mouse eyes were fixed with 10% formalin and eyecups were obtained by removing the anterior poles and neurosensory retina. Eyecups containing the RPE-choroid-sclera complex were blocked with PBS containing 1% BSA and 0.5% triton X-100 and then incubated with 10 μg/mL of FITC-isolectin B4 overnight at 4°C. Fluorescent images captured using the 20× objective of a scanning confocal microscope were analyzed. On both days 7 and 14, the sizes of CNV volumes were much smaller in TSA-treated mice than in mice that received PBS only. (*: t test p<0.05, **: t test p<0.005; n = 10/group) (bar = 50 μm).

To study the effect of TSA on the development of laser-induced CNV, the formation of CNV was evaluated by three methods: fluorescein angiography (FA), histology and measurement of CNV volume. 5 drug-naïve mice per group were administered intraperitoneal injection of 20 mg/kg of TSA or the same volume of sterile PBS immediately after laser photocoagulation and every 48 h thereafter for 7 or 14 days. Late-phase FAs of both eyes in each mouse from the control and TSA-treated groups were evaluated according to the grading system described in the Materials and Methods section [53]. Ten animals, 20 eyes and 34 lesions were examined in the control group, and 10 animals, 20 eyes and 35 lesions were examined in the TSA group. Representative angiographic images in control and TSA-treated mice on days 7 and 14 are shown in Fig. 11A. TSA-treated mice exhibited attenuated CNV formation and leakage compared with PBS-treated mice. Mice treated with TSA demonstrated a significant reduction of FA score compared to the control animals on days 7 and 14 after laser photocoagulation (Fig. 11B; p<0.05). For histologic analysis, 8 animals, 8 eyes and 8 lesions were examined in the control group, and 9 animals, 9 eyes and 9 lesions were examined in the TSA group. TSA treatment resulted in statistically significant reductions in lesion area on days 7 and 14 after laser photocoagulation (Fig. 12A-B; p<0.05). Choroidal flatmount with fluorescein-conjugated isolectin B4 staining was used to assess the volume of the CNV lesions on days 7 and 14 after laser photocoagulation. Ten animals, 10 eyes and 20 lesions were examined in the control group, and 10 animals, 10 eyes and 28 lesions were examined in the TSA group. Mice treated with TSA demonstrated a significantly smaller lesion size compared to those treated with PBS (Fig. 13A). Quantitative measurement of the volumes of the CNV lesions showed that TSA treatment resulted in an approximately 65.5% and 65.9% reduction, respectively, in choroidal vascular volume, compared with that of control mice, 1 week and 2 weeks after laser photocoagulation (Fig. 13B; p<0.05).

**Fig 14:**
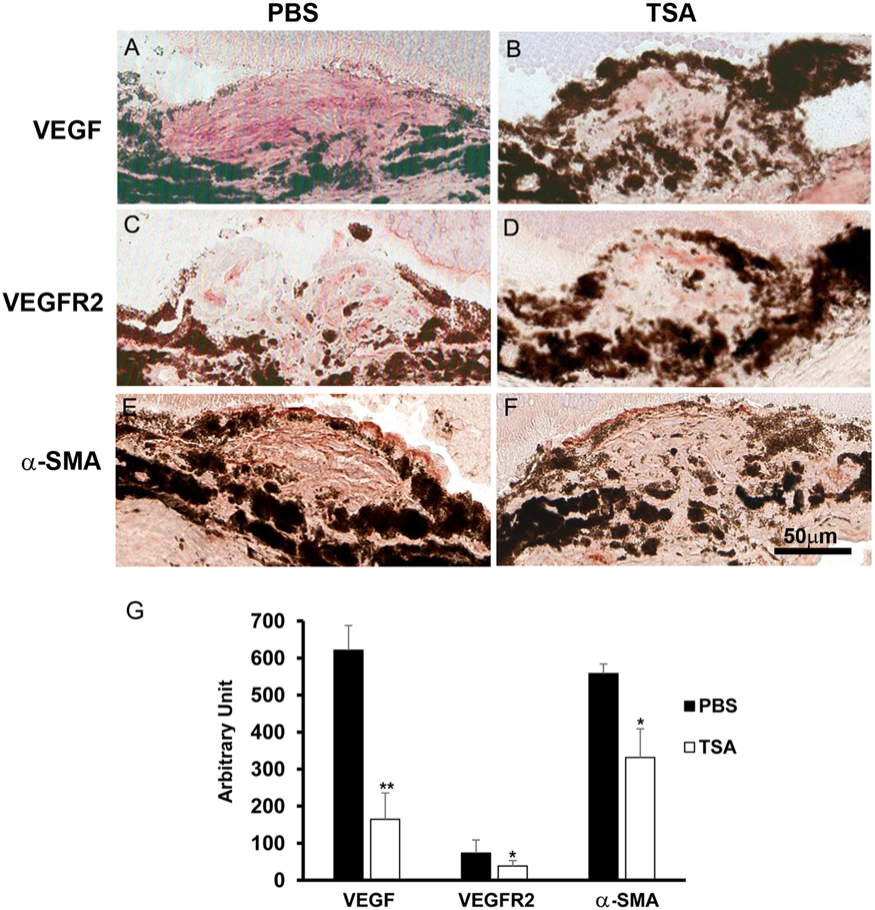
TSA reduces VEGF, VEGFR2 and α-SMA in mouse CNV lesions. Immunohistochemical staining was performed on murine retinal cryostat sections in CNV lesions day 7 post-laser for (A-B) VEGF, (C-D) VEGFR2 and (E-F) α-SMA. Figures on the left panel (A, C and E) are from PBS control mice, and figures on the right panel (B, D and F) are from TSA-treated mice. TSA reduced the amount of cells stained positively for (A) VEGF, (C) VEGFR2 and (E) α-SMA, when compared to PBS controls (B, D and F). (G) Quantification of positively stained area for each protein normalized by the size of the CNV lesions. (*: t test p<0.05; **: t test p<0.01; n = 4/group; bar = 50 μm).

TSA regulates VEGF, VEGFR2 and α-SMA expression in CNV mouse model Our *in vitro* experiment suggests that TSA inhibits angiogenesis by down-regulating VEGF and up-regulating PEDF in RPE cells, as well as down-regulating VEGFR2 in HUVECs (Figs. 5A and B; 10A and B). TSA suppresses RPE activation by down-regulating TGF-β-induced α-SMA as well (Fig. 4A and B). A similar pattern was observed by immunostaining in our CNV mouse model (Fig. 14). Three animals, 3 eyes, and 4 lesions were examined in the control group from day 7, and 3 animals, 4 eyes, and 5 lesions were examined in the TSA group from day 7. Lower levels of VEGF, VEGFR2 and α-SMA were expressed in the CNV lesions of TSA-treated mice, in comparison to PBS-treated controls (Fig. 14A-G;). Since PEDF is rapidly secreted once it is synthesized in RPE cells, we were unable to capture its presence by immunostaining. The regulation of these proteins by TSA in our mouse model indicates that modulation of angiogenic and EMT gene expression is one route that TSA inhibits CNV formation in mice.

### TSA does not induce apoptosis in normal choroidal vasculature

To investigate whether TSA has any toxicity on normal choroidal vasculature, we performed TUNEL assay on mice that had received either PBS or TSA for 14 days, in the absence of laser photocoagulation, as well as in cryosections from the laser CNV mice. Flatmounts from three mice and six eyes were examined in the control group and three mice and six eyes were examined in the TSA group. Cryosections from three animals and three eyes were each examined in each of the PBS and TSA groups. No TUNEL-positive signal was observed in the normal choroidal vasculature in any flatmount or cryosection from either the control or the TSA group while DNase-treated positive control sections showed extensive nuclear positivity (results not shown).

## Discussion

Inhibition of VEGF is the focus of CNV therapies that include bevacizumab, ranibizumab and pegaptanib [13]. However, these drugs must be dosed intravitreally, given early in the course of the disease; they must be administered periodically; and can only partially restore vision [13]. Most of the preclinical approaches for therapy target only one aspect of the pathogenesis [13]. Here, we demonstrated that TSA has a multi-faceted anti-angiogenic effect in RPE and ECs. We propose that TSA or related agents should be considered for their potential as an efficacious treatment option for CNV.

Inhibition of angiogenesis by HDACi has been demonstrated in cancer biology and other physiological systems [41–43; 45–47]. TSA is the prototypic hydroxamic acid HDACi [51] that regulates the activity of Classes I and II HDACs [56]. The role of HDACi’s in regulating CNV formation has been previously reported but not evaluated in sufficient mechanistic detail. The synthetic HDACi *N*-hydroxy-7-(2-naphthylthio) heptanomide (HNHA) inhibits laser-induced CNV formation in mice [48]. Kim *et al*. also showed that HNHA impedes VEGF-induced tube formation and suppresses the proliferation of HUVECs, but the effect of TSA on RPE’s role in promoting CNV formation was not examined [48]. Deroanne *et al*. studied TSA’s and SAHA’s anti-angiogenic effect in various angiogenesis assays, and established that TSA down-regulated the expression of the VEGF receptors in HUVECs [57]. However, they did not explore the possible epigenetic modulation of these genes via ChIP assay or the effect of these HDACi’s in VEGFR2 activation [57]. Iordache *et al*. investigated the role of HDAC in ECs. The authors stated that mature ECs have lower angiogenic potential; on the other hand, endothelial progenitor cells (EPC) may play a significant role in vascular healing and repair. They found that HDACs participate in EPC’s differentiation into EC [58]. As a critical supportive cell type of the retina and the choroid, [22–28] we found that TSA inhibits RPE cells' proliferation, activation, and expression of VEGF and HIF-1α. We also showed that angiogenesis can be further diminished by an HDACi through the up-regulation of the anti-angiogenic and neuro-protective PEDF. We then showed that TSA reduces CEC's proliferation and survival, and induces cell death, by regulating cell signaling. Furthermore, TSA dramatically reduces the expression of a major angiogenesis-related cell surface receptor, VEGFR2, in CEC. We demonstrated that RPE cells' angiogenic potential diminished by TSA may lead to an anti-angiogenic effect in CEC, consequently resulting in inhibition of CNV.

CNV-associated wound healing requires the expression of various growth factors [14] that initiate EMT and RPE proliferation [59]. VEGF co-localizes with α-SMA-positive transdifferentiated RPE cells [5], and quiescent, monolayered RPE cells become multilayered in human CNV membranes [60]. We found that TSA arrested RPE cell cycle at G1 phase, likely inhibiting RPE proliferation by inducing the cell cycle kinase inhibitors p21 and p15 [61]. TGF-β, the inhibition of which reduces laser-induced CNV formation in rats [62], causes EMT in RPE cells [63] by inducing the major EMT-associated transcription factor SNAIL [15]. We showed that TSA suppressed the TGF-β-induced α-SMA, an EMT marker [15], in primary human RPE cells and in laser-induced mouse CNV cryosections. Xiao *et al*. also demonstrated that TSA inhibits RPE cell proliferation, suppresses RPE cell cycle progression at G1, and reduces the up-regulation of α-SMA in response to TGF-β, but this was performed in an immortalized RPE cell line (ARPE-19) which may not reflect the physiological state of native RPE as well as the low passage primary RPE cells used in the present study [64]. PDGF is expressed in CNV-associated wound healing [14], the concurrent inhibition of which with VEGF attenuates CNV formation [65] and is shown to inhibit cell motility [66–68]. TSA impeded PDGF-induced RPE cell migration, while promoting RPE cell attachment to fibronectin. We propose that the pro-cell attachment effect of TSA on RPE cells opposes RPE cell migration induced by PDGF. The inhibition of RPE cell migration elicited by TSA is unlikely to be due to TSA’s anti-proliferative effect, as the duration of the migration assay lasted only 5 hrs.

The expression of VEGF is increased in CNV [69], while PEDF expression is reduced in AMD retinas [70]. We showed that TSA reduced HIF-1α and VEGF protein levels and up-regulated PEDF in RPE cells. However, at 0.1 μM and 0.3 μM TSA, HIF-1α protein levels were even higher than that in cells treated with CoCl_2_ only, while VEGF protein levels gradually decreased from 0.05 μM to 0.5 μM TSA. HIF-1α is regulated by acetylation/deacetylation and CoCl_2_. [71–74]. In hypoxia, the acetylation of HIF-1α at lysine 709 by p300, which can be blocked by HDAC1, prevents the polyubiquitination and subsequent proteasomal degradation of HIF-1α. [71] CoCl_2_ mimics hypoxia by binding the oxygen-dependent degradation domain in HIF-1α which blocks its interaction with the von Hippel-Lindau protein [72]. HDAC6 mediates the association of HIF-1α with heat shock protein 90, resulting in HIF-1α’s degradation via the proteasome [73]. HDACs activities may be differentially regulated at different TSA concentrations. HDAC1 and HDAC6 may be inhibited at lower TSA concentrations, causing CoCl_2_’s potentiation of HIF-1α to associate with p300 that further preserves HIF-1α’s stability. Other mechanisms may be activated at higher TSA concentrations. HDAC4 deacetylates HIF-1α at its five amino-terminal lysine residues in hypoxic and CoCl_2_-treated cells, which preserves HIF-1α’s stability and increases its transcriptional activity. [74] TSA may target HDAC4 at higher concentrations and reduce HIF-1α protein level. TSA reduced active transcription at VEGF’s promoter, suggesting that TSA’s inhibition of VEGF may be a direct epigenetic effect, possibly by inducing compaction of chromatin around the hypoxia response element of the VEGF promoter [75]. Conversely, the reduced HIF-1α protein levels after TSA treatment may abrogate the induction of VEGF expression as well. TSA exerted a statistically significant enhancement of PEDF transcription, indicating that epigenetic mechanism may be a component in the effect of TSA on PEDF expression. Interestingly, plasminogen kringle 5 (K5) reduced the levels of HIF-1α and VEGF while PEDF was up-regulated in an ischemic retinal neovascularization rat model. HIF-1α failed to translocate to the nucleus in the presence of K5, suggesting the increased PEDF expression is related to suppression of HIF-1α target genes. [76] In bovine retinal capillary endothelial cells treated with VEGF, the expression and secretion of PEDF was significantly reduced. [77] TSA’s up-regulation of PEDF may be caused by the reduced HIF-1α and VEGF protein levels. Neovascularization in CNV is comparable to tumor angiogenesis that can be epigenetically regulated [45–46, 78–79]. The inhibitory effect of HDACi’s on carcinogenic angiogenesis implicates a common theme of targeting the HIF-1α-VEGF-PEDF axis, which may point to a new therapeutic approach in treating CNV.

HDACi’s can modulate EC proliferation and functions. We found that TSA inhibited BCEC survival as shown in the TUNEL assay, perhaps targeting cells that are multiplying and engaging in angiogenesis. TSA appeared to impede the proliferation and survival of BCECs by decreasing phosphorylated p42/44, which directs cell proliferation in the presence of growth stimuli [80]. Simultaneously, TSA reduced the amount of pro-survival [81] and cyclin D1-preserving [82] phosphorylated Akt. Histone acetyltransferase (HAT) activity may require or result in the modulation of signaling pathways. TNF activates and induces the nuclear translocation of Akt that in turn phosphorylates the HAT p300, resulting in the up-regulation of ICAM-1 [83]. In hypoxia, p42/44 promotes the transactivation potential of p300 and its association with HIF-1α [84], mediating HIF-1α-induced gene expression. Furthermore, p38 and Akt are required for recruiting and then degrading p300 in nucleotide excision repair [85]. Hence, regulation of cell signaling is involved in modulating HAT-mediated pathways.

TSA activated caspase 3 in EC at higher concentrations, echoing our results in the TUNEL assay. Active caspase 3 brings about the irreversible process of cell death [86], and HDACi’s induce apoptosis through the intrinsic pathway [56]. TSA also inhibited *in vitro* angiogenesis in HUVECs in the presence of VEGF. Neovascularization requires ECs to proliferate and survive [12]. The inhibition of p42/44 and Akt, the activation of caspase 3, and the increased amount of TUNEL-positive BCECs caused by TSA hinder BECE’s angiogenic potential, which could explain the result of our *in vitro* angiogenesis assay. Previous studies showed that TSA impeded angiogenesis in embryoid bodies and sprouting from rat aortic rings, and reduced the mRNA expression of VEGFR1, VEGFR2, Neuropilin-1, TIMP-1 and MMP-1 in HUVECs. Taken together, HDACi’s exert a broad-spectrum anti-angiogenic effect on ECs [87]. Despite the induction of cell death in EC *in vitro*, 14 days of treatment with TSA *in vivo* elicited no TUNEL+ cell death in the quiescent, normal choroidal vasculature (results not shown).

To stimulate angiogenesis, VEGF binds and activates VEGFR2, VEGF’s major pro-angiogenic receptor, to promote the survival, proliferation and migration of ECs [88]. Macrovascular EC may in some cases respond differently to stimuli when compared to microvascular EC [89, 90]. It would have been best to perform all studies with BCECs, but their supply was limited and it was necessary that some experiments were performed using HUVECs. In this study, VEGFR2 was prominently down-regulated by TSA in both microvascular BCECs and macrovascular HUVECs, and previous studies have shown that VEGFR2 is phosphorylated in response to VEGF exposure in both HUVECs and BCECs [89, 91]. The down-regulation of VEGFR2 by inhibiting histone deacetylation seems counterintuitive. HOXA9, a transcription factor that promotes angiogenic gene expression [92] and induces VEGFR2, is down-regulated by HDACi exposure, causing reduced VEGFR2 levels [93]. Furthermore, the gene-activating ALL-1 histone methyltransferase complex containing HDACs 1 and 2 is present on HOXA9’s promoter. Nakamura *et al*. suggests that ALL-1’s activity may require histone deacetylation [93]. Thus, the regulation of an upstream gene by epigenetics may explain our result on VEGFR2. On the other hand, HDACs 6 and 10 can induce the depletion of VEGFR2 via heat shock proteins [94]; TSA may suppress VEGFR2 by additional non-epigenetic mechanisms. Further, VEGF-induced phosphorylation of VEGFR2 and its downstream signaling was inhibited by TSA in HUVECs. Since VEGF mediates angiogenesis through its activation of VEGFR2-directed gene expression, our result suggests that this HDACi can inhibit the survival, mitogenicity and motogenicity of ECs in the pathogenesis of CNV. These results mirror other studies of the VEGF receptors. With either of two HDACi’s, SAHA or LAQ824, both VEGFR1 and VEGFR2 were down-regulated in the human colon cancer cell line HCT116 [57]. Intriguingly, VEGFR1 and several other pro-angiogenic genes are up-regulated in fibroblast growth factor 2- and epidermal growth factor-treated mouse yolk sac endothelial cells (YSECs) and HUVECs in conjunction with an increase of histone H3 lysine 56 acetylation mediated by the histone chaperone, HIRA. However, this pro-angiogenic effect is probably caused by an HDAC-independent mechanism and likely stimulus-specific, because H3acK56 incorporation and VEGFR1 expression are not restored by exposing HIRA-knocked down YSECs to a HDACi. [95].

TSA significantly attenuated CNV formation in our laser-induced mouse model. CNV leakage, as shown in FA analysis, as well as CNV volume, was much less prominent in TSA-treated mice, compared to controls. As discussed above, TSA exhibits its anti-angiogenic activity *in vitro* in RPE cells, BCECs and HUVECs, which likely results in its inhibiting angiogenesis *in vivo*. Indeed, immunohistochemical staining of retinal sections from our mouse model demonstrated that TSA inhibited the expression of VEGF, VEGFR2 and SMA in CNV lesions. As shown by Crosson *et al*., TSA protected rats from ischemic retinal injury and reduced metalloproteinase (MMPs) secretion by blocking the effect of TNF [40]. This implies that TSA can reduce the hypoxic and inflammatory response in the eye and arrest CNV formation. TSA can also potentially minimize retinal injury and vision loss at the involution stage of CNV. Scarring that occurs in end stage CNV is caused by the pro-fibrotic TGF-β and the ECM-modifying tissue inhibitors of matrix metalloproteinases in activated RPE cells. [96] Yet photoreceptors would be blocked by the scar tissue from receiving nutrients from the choroid and die, exacerbating vision loss [97]. We showed that inhibiting HDACs subdues the wound-healing response, which likely suppresses fibrosis. HDAC inhibition also promotes MMP expression [98], and may thus lessen the loss of photoreceptors. Suppression of wound healing by a HDACi through the inhibition of TGFβ-1-induced SMA and fibronectin has also been illustrated in corneal fibroblasts [99]. Taken together, TSA exerts a pronounced anti-angiogenic effect that can attenuate CNV formation. Future studies should be undertaken to evaluate the potential efficacy of class-specific HDACi’s to minimize unforeseen off-target effects of broad-spectrum HDACi’s, as well as the clinically approved HDACi’s such as SAHA as potential therapy of CNV.

## References

[1] Zarbin MA. Current concepts in the pathogenesis of age-related macular degeneration. Arch Ophthalmol. 2004;122:598–614. 10.1001/archopht.122.4.598 15078679

[2] Aiello LP, Northrup JM, Keyt BA, Takagi H, Iwamoto MA. Hypoxic regulation of vascular endothelial growth factor in retinal cells. Arch Ophthalmol. 1995;113:1538–1544. 7487623 10.1001/archopht.1995.01100120068012

[3] Amin R, Puklin J, Frank RN. Growth factor localization in choroidal neovascular membranes of age-related macular degeneration. Invest Ophthalmol Vis Sci. 1994;35:3178–3188. 7519180

[4] Frank RN, Amin RH, Eliott D, Puklin JE, Abrams GW. Basic fibroblast growth factor and vascular endothelial growth factor are present in epiretinal and choroidal neovascular membranes. Am J Ophthalmol. 1996;122:393–403. 8794712 10.1016/s0002-9394(14)72066-5

[5] Lopez PF, Sippy BD, Lambert HM, Thach AB, Hinton DR. Transdifferentiated retinal pigment epithelial cells are immunoreactive for vascular endothelial growth factor in surgically excised age-related macular degeneration-related choroidal neovascular membranes. Invest Ophthalmol Vis Sci. 1996;37:855–868. 8603870

[6] Zhang NL, Samadani EE, Frank RN. Mitogenesis and retinal pigment epithelial cell antigen expression in the rat after krypton laser photocoagulation. Invest Ophthalmol Vis Sci. 1993;34:2412–2424. 8325749

[7] Elner VM, Strieter RM, Elner SG, Baggiolini M, Lindley I, Kunkel SL. Neutrophil chemotactic factor (IL-8) gene expression by cytokine-treated retinal pigment epithelial cells. Am J Pathol. 1990;136:745–750. 2183623 PMC1877641

[8] Oh H, Takagi H, Takagi C, Suzuma K, Otani A, Ishida K, et al The potential angiogenic role of macrophages in the formation of choroidal neovascular membranes. Invest Ophthalmol Vis Sci. 1999;40:1891–1898. 10440240

[9] Majka S, McGuire PG, Das A. Regulation of matrix metalloproteinase expression by tumor necrosis factor in a murine model of retinal neovascularization. Invest Ophthalmol Vis Sci. 2002;43:260–266. 11773040

[10] Das A, McLamore A, Song W, McGuire PG. Retinal neovascularization is suppressed with a matrix metalloproteinase inhibitor. Arch Ophthalmol. 1999;117:498–503. 10206578 10.1001/archopht.117.4.498

[11] Ogata N, Ando A, Uyama M, Matsumura M. Expression of cytokines and transcription factors in photocoagulated human retinal pigment epithelial cells. Graefes Arch Clin Exp Ophthalmol. 2001;239:87–95. 11372550 10.1007/s004170000235

[12] Ferrara N, Davis-Smyth T. The biology of vascular endothelial growth factor. Endocr Rev. 1997;18:4–24. 9034784 10.1210/edrv.18.1.0287

[13] Bressler SB. Introduction: Understanding the role of angiogenesis and antiangiogenic agents in age-related macular degeneration. Ophthalmology 2009;116(10 Supp.):S1–7. 10.1016/j.ophtha.2009.06.045 19800534

[14] Hirasawa M, Noda K, Noda S, Suzuki M, Ozawa Y, Shinoda K, et al Transcriptional factors associated with epithelial-mesenchymal transition in choroidal neovascularization. Mol Vis. 2011;17:1222–30. 21617757 PMC3102030

[15] Schlingemann RO. Role of growth factors and the wound healing response in age-related macular degeneration. Graefes Arch Clin Exp Ophthalmol. 2004;242:91–101. 10.1007/s00417-003-0828-0 14685874

[16] Saika S, Yamanaka O, Flanders KC, Okada Y, Miyamoto T, Sumioka T, et al Epithelial-mesenchymal transition as a therapeutic target for prevention of ocular tissue fibrosis. Endocr Metab Immune Disord Drug Targets. 2008;8:69–76. 10.2174/187153008783928343 18393925

[17] Watanabe D, Takagi H, Suzuma K, Oh H, Ohashi H, Honda Y. Expression of connective tissue growth factor and its potential role in choroidal neovascularization. Retina. 2005;25:911–8. 16205572 10.1097/00006982-200510000-00015

[18] Ogata N, Yamamoto C, Miyashiro M, Yamada H, Matsushima M, Uyama M. Expression of transforming growth factor-beta mRNA in experimental choroidal neovascularization. Curr Eye Res. 1997;16:9–18. 9043818 10.1076/ceyr.16.1.9.5121

[19] Nagineni CN, Samuel W, Nagineni S, Pardhasaradhi K, Wiggert B, Detrick B, et al Transforming growth factor-beta induces expression of vascular endothelial growth factor in human retinal pigment epithelial cells: involvement of mitogen-activated protein kinases. J Cell Physiol. 2003;197:453–62. 10.1002/jcp.10378 14566975

[20] Fong GH. Regulation of angiogenesis by oxygen sensing mechanisms. J Mol Med 2009;87:549–560. 10.1007/s00109-009-0458-z 19288062

[21] Grierson I, Hiscott P, Hogg P, Robey H, Mazure A, Larkin G. Development, repair and regeneration of the retinal pigment epithelium. Eye 1994;8:255–262. 10.1038/eye.1994.54 7525361

[22] Korte GE, Reppucci V, Henkind P. RPE destruction causes choriocapillary atrophy. Invest Ophthalmol Vis Sci. 1984;25:1135–1145. 6480292

[23] Sakamoto T, Sakamoto H, Murphy TL, Spee C, Soriano D, Ishibashi T, et al Vessel formation by choroidal endothelial cells in vitro is modulated by retinal pigment epithelial cells. Arch Ophthalmol. 1995;113:512–520. 10.1001/archopht.1995.01100040134039 7536000

[24] Castellarin AA, Nasir M, Sugino IK, Zarbin MA. Progressive presumed choriocapillaris atrophy after surgery for age-related macular degeneration. Retina 1998;18:143–149. 9564695 10.1097/00006982-199818020-00008

[25] Blaauwgeers HG, Holtkamp GM, Rutten H, Witmer AN, Koolwijk P, Partanen TA, et al Polarized vascular endothelial growth factor secretion by human retinal pigment epithelium and localization of vascular endothelial growth factor receptors on the inner choriocapillaris. Evidence for a trophic paracrine relation. Am J Pathol. 1999;155:421–428. 10.1016/S0002-9440(10)65138-3 10433935 PMC1866848

[26] Sugasawa K, Deguchi J, Okami T, Yamamoto A, Omori K, Uyama M, et al Immunocytochemical analyses of distributions of Na, K-ATPase and GLUT1, insulin and transferrin receptors in the developing retinal pigment epithelial cells. Cell Struct Funct. 1994;19:21–28. 8069944 10.1247/csf.19.21

[27] Bergersen L, Jóhannsson E, Veruki ML, Nagelhus EA, Halestrap A, Sejersted OM, et al Cellular and subcellular expression of monocarboxylate transporters in the pigment epithelium and retina of the rat. Neuroscience 1999;90:319–331. pii: S0306-4522(98)00427-8 10188957 10.1016/s0306-4522(98)00427-8

[28] McLeod DS, Grebe R, Bhutto I, Merges C, Baba T, Lutty GA. Relationship between RPE and choriocapillaris in age-related macular degeneration. Invest Ophthalmol Vis Sci. 2009;50:4982–91. 10.1167/iovs.09-3639 19357355 PMC4829357

[29] Arjamaa O, Nikinmaa M. Oxygen-dependent diseases in the retina: role of hypoxia-inducible factors. Exp Eye Res 2006;83:473–483 16750526 10.1016/j.exer.2006.01.016

[30] Penn JS, Madan A, Caldwell RB, Bartoli M, Caldwell RW, Hartnett ME. Vascular endothelial growth factor in eye disease. Prog Retin Eye Res. 2008;27:331–371. 10.1016/j.preteyeres.2008.05.001 18653375 PMC3682685

[31] Grossniklaus H. E., Kang S. J., & Berglin L. Animal Models of Choroidal and Retinal Neovascularization. Progress in Retinal and Eye Research 2010;29:500–519. 10.1016/j.preteyeres.2010.05.003 20488255 PMC2962694

[32] Ishibashi T, Hata Y, Yoshikawa H, Nakagawa K, Sueishi K, Inomata H. Expression of vascular endothelial growth factor in experimental choroidal neovascularization. Graefes Arch Clin Exp Ophthalmol. 1997;235:159–167. 9085111 10.1007/BF00941723

[33] Gehlbach P, Demetriades AM, Yamamoto S, Deering T, Duh EJ, Yang HS, et al Periocular injection of an adenoviral vector encoding pigment epithelium-derived factor inhibits choroidal neovascularization. Gene Ther. 2003;10:637–646. 10.1038/sj.gt.3301931 12692592

[34] Taniwaki T, Becerra SP, Chader GJ, Schwartz JP. Pigment epithelium-derived factor is a survival factor for cerebellar granule cells in culture. J Neurochem. 1995;64:2509–2517. 7760030 10.1046/j.1471-4159.1995.64062509.x

[35] Sonoda S, Sreekumar PG, Kase S, Spee C, Ryan SJ, Kannan R, et al Attainment of polarity promotes growth factor secretion by retinal pigment epithelial cells: relevance to age-related macular degeneration. Aging 2009;2:28–42. 20228934 10.18632/aging.100111 PMC2837203

[36] Jablonski MM, Tombran-Tink J, Mrazek DA, Iannaccone A. Pigment epithelium-derived factor supports normal development of photoreceptor neurons and opsin expression after retinal pigment epithelium removal. J Neurosci. 2000;20:7149–7157. 11007870 10.1523/JNEUROSCI.20-19-07149.2000 PMC6772781

[37] Cayouette M, Smith SB, Becerra SP, Gravel C. Pigment epithelium-derived factor delays the death of photoreceptors in mouse models of inherited retinal degenerations. Neurobiol Dis. 1999;6:523–532. 10.1006/nbdi.1999.0263 10600408

[38] Cao W, Tombran-Tink J, Elias R, Sezate S, Mrazek D, McGinnis JF. In vivo protection of photoreceptors from light damage by pigment epithelium-derived factor. Invest Ophthalmol Vis Sci. 2001;42:1646–1652. 11381073

[39] Dawson DW, Volpert OV, Gillis P, Crawford SE, Xu H, Benedict W, et al Pigment epithelium-derived factor: a potent inhibitor of angiogenesis. Science 1999;285:245–248. 10398599 10.1126/science.285.5425.245

[40] Gao G, Li Y, Zhang D, Gee S, Crosson C, Ma J. Unbalanced expression of VEGF and PEDF in ischemia-induced retinal neovascularization. FEBS Lett. 2001;489:270–276. pii: S001457930102110X 11165263 10.1016/s0014-5793(01)02110-x

[41] Zgouras D, Becker U, Loitsch S, Stein J. Modulation of angiogenesis-related protein synthesis by valproic acid. Biochem Biophys Res Commun. 2004;316:693–697. 10.1016/j.bbrc.2004.02.105 15033455

[42] Sasakawa Y, Naoe Y, Noto T, Inoue T, Sasakawa T, Matsuo M, et al Antitumor efficacy of FK228, a novel histone deacetylase inhibitor, depends on the effect on expression of angiogenesis factors. Biochem Pharmacol. 2003;66:897–906. pii: S0006295203004118 12963476 10.1016/s0006-2952(03)00411-8

[43] Chou CW, Chen C. HDAC inhibition upregulates the expression of angiostatic ADAMTS1. FEBS Lett. 2008;582:4059–4065. 10.1016/j.febslet.2008.10.048 19007777

[44] Vaissière T, Sawan C, Herceg Z. Epigenetic interplay between histone modifications and DNA methylation in gene silencing. Mutat Res. 2008;659:40–48. 10.1016/j.mrrev.2008.02.004 18407786

[45] Mottet D, Bellahcène A, Pirotte S, Waltregny D, Deroanne C, Lamour V, et al Histone deacetylase 7 silencing alters endothelial cell migration, a key step in angiogenesis. Circ Res. 2007;101:1237–1246. 10.1161/CIRCRESAHA.107.149377 17947801

[46] Crosson CE, Mani SK, Husain S, Alsarraf O, Menick DR. Inhibition of histone deacetylase protects the retina from ischemic injury. Invest Ophthalmol Vis Sci. 2010;51:3639–3645. 10.1167/iovs.09-4538 20164449 PMC2904015

[47] Fan J, Alsarraf O, Dahrouj M, Platt KA, Chou CJ, Rice DS, et al Inhibition of HDAC2 protects the retina from ischemic injury. Invest Ophthalmol Vis Sci. 2013;54:4072–4080. 10.1167/iovs.12-11529 23696608 PMC3681476

[48] Kim JH, Kim JH, Oh M, Yu YS, Kim KW, Kwon HJ. N-hydroxy-7-(2-naphthylthio) heptanomide inhibits retinal and choroidal angiogenesis. Mol Pharm. 2009;6:513–519. 10.1021/mp800178b 19718802

[49] Sonoda S, Spee C, Barron E, Ryan SJ, Kannan R, Hinton DR. A protocol for the culture and differentiation of highly polarized human retinal pigment epithelial cells. Nat Protoc. 2009;4:662–673. 10.1038/nprot.2009.33 19373231 PMC2688697

[50] Hoffmann S, Spee C, Murata T, Cui JZ, Ryan SJ, Hinton DR. Rapid isolation of choriocapillary endothelial cells by Lycopersicon esculentum-coated Dynabeads. Graefes Arch Clin Exp Ophthalmol. 1998;236:779–84. 9801894 10.1007/s004170050158

[51] He S, Ding Y, Zhou J, Krasnoperov V, Zozulya S, Kumar SR. Soluble EphB4 regulates choroidal endothelial cell function and inhibits laser-induced choroidal neovascularization.Invest Ophthalmol Vis Sci. 2005;46:4772–9. 10.1167/iovs.05-0502 16303978

[52] He PM, He S, Garner JA, Ryan SJ, Hinton DR. Retinal pigment epithelial cells secrete and respond to hepatocyte growth factor. Biochem Biophys Res Commun. 1998;249:253–257. 10.1006/bbrc.1998.9087 9705867

[53] Iruela-Arispe ML, Bornstein P, Sage H. Thrombospondin exerts an antiangiogenic effect on cord formation by endothelial cells in vitro. Proc Natl Acad Sci U S A 1991;88:5026–5030. 1711216 10.1073/pnas.88.11.5026 PMC51800

[54] Sreekumar PG, Zhou J, Sohn J, Spee C, Ryan SJ, Maurer BJ, et al N-(4-hydroxyphenyl) retinamide augments laser-induced CNV in mice. Invest Ophthalmol Vis Sci. 2008;49:1210–1220. 10.1167/iovs.07-0667 18326751

[55] Qian DZ, Kachhap SK, Collis SJ, Verheul HM, Carducci MA, Atadja P, et al Class II histone deacetylases are associated with VHL-independent regulation of hypoxia-inducible factor 1 alpha. Cancer Res. 2006;66:8814–8821. 10.1158/0008-5472.CAN-05-4598 16951198

[56] Aurora AB, Biyashev D, Mirochnik Y, Zaichuk TA, Sánchez-Martinez C, Renault MA, et al NF-kappaB balances vascular regression and angiogenesis via chromatin remodeling and NFAT displacement. Blood 2010;116:475–484. 10.1182/blood-2009-07-232132 20203265 PMC2913457

[57] Deroanne CF, Bonjean K, Servotte S, Devy L, Colige A, Clausse N, et al Histone deacetylases inhibitors as anti-angiogenic agents altering vascular endothelial growth factor signaling. Oncogene 2002;21:427–436. 11821955 10.1038/sj.onc.1205108

[58] Iordache F, Buzila C, Constantinescu A, Andrei E, Maniu H. Histone deacetylase (HDAC) inhibitors down-regulate endothelial lineage commitment of umbilical cord blood derived endothelial progenitor cells. Int J Mol Sci. 2012;13:15074–85. 10.3390/ijms131115074 23203112 PMC3509628

[59] Tamiya S, Liu L, Kaplan HJ (2010) Epithelial-mesenchymal transition and proliferation of retinal pigment epithelial cells initiated upon loss of cell-cell contact. Invest Ophthalmol Vis Sci. 51:2755–63. 10.1167/iovs.09-4725 20042656

[60] Seregard S, Algvere PV, Berglin L. Immunohistochemical characterization of surgically removed subfoveal fibrovascular membranes. Graefes Arch Clin Exp Ophthalmol. 1994;232:325–9. 10.1007/BF00175983 8082839

[61] Codd R, Braich N, Liu J, Soe CZ, Pakchung AA. Zn(II)-dependent histone deacetylase inhibitors: suberoylanilide hydroxamic acid and trichostatin A. Int J Biochem Cell Biol. 2009;41:736–9. 10.1016/j.biocel.2008.05.026 18725319

[62] Recalde S, Zarranz-Ventura J, Fernández-Robredo P, García-Gómez PJ, Salinas-Alamán A, Borrás-Cuesta F, et al Transforming growth factor-β inhibition decreases diode laser-induced choroidal neovascularization development in rats: P17 and P144 peptides. Invest Ophthalmol Vis Sci. 2011;52:7090–7. 10.1167/iovs.11-7300 21810978

[63] Gamulescu MA, Chen Y, He S, Spee C, Jin M, Ryan SJ, et al Transforming growth factor beta2-induced myofibroblastic differentiation of human retinal pigment epithelial cells: regulation by extracellular matrix proteins and hepatocyte growth factor. Exp Eye Res. 2006;83:212–22. 10.1016/j.exer.2005.12.007 16563380

[64] Xiao W, Chen X, Liu X, Luo L, Ye S, Liu Y. Trichostatin A, a histone deacetylase inhibitor, suppresses proliferation and epithelial-mesenchymal transition in retinal pigment epithelium cells. J Cell Mol Med. 2014;18:646–55. 10.1111/jcmm.12212 24456602 PMC4000116

[65] Kwak N, Okamoto N, Wood JM, Campochiaro PA. VEGF is major stimulator in model of choroidal neovascularization. Invest Ophthalmol Vis Sci. 2000;41:3158–64. 10967078

[66] Yao Q, Renault MA, Chapouly C, Vandierdonck S, Belloc I, Jaspar-Vinassa B, et al Sonic hedgehog mediates a novel pathway of PDGF-BB-dependent vessel maturation. Blood. 2014; 123:2429–37. 10.1182/blood-2013-06-508689 24472833

[67] Bartok B, Hammaker D, Firestein GS. Phosphoinositide 3-Kinase δ Regulates Migration and Invasion of Synoviocytes in Rheumatoid Arthritis. J Immunol. 2014;192:2063–70. 10.4049/jimmunol.1300950 24470496

[68] Huang J, Xie LD, Luo L, Zheng SL, Wang HJ, Xu CS. Silencing heat shock protein 27 (HSP27) inhibits the proliferation and migration of vascular smooth muscle cells in vitro. Mol Cell Biochem. 2014;390:115–21. 10.1007/s11010-014-1962-1 24469469

[69] Sheridan CM, Pate S, Hiscott P, Wong D, Patwell DM, Kent D. Expression of hypoxia-inducible factor-1alpha and -2alpha in human choroidal neovascular membranes. Graefes Arch Clin Exp Ophthalmol. 2009;247:1361–1367. 10.1007/s00417-009-1133-3 19590888

[70] Tong JP, Yao YF. Contribution of VEGF and PEDF to choroidal angiogenesis: a need for balanced expressions. Clin Biochem. 2006;39:267–276. 10.1016/j.clinbiochem.2005.11.013 16409998

[71] Geng H, Liu Q, Xue C, David LL, Beer TM, Thomas GV, et al HIF1α protein stability is increased by acetylation at lysine 709. J Biol Chem. 2012;287:35496–35505. 10.1074/jbc.M112.400697 22908229 PMC3471753

[72] Yuan Y, Hilliard G, Ferguson T, Millhorn DE. Cobalt inhibits the interaction between hypoxia-inducible factor-alpha and von Hippel-Lindau protein by direct binding to hypoxia-inducible factor-alpha. J Biol Chem. 2003;278:15911–15916. 10.1074/jbc.M300463200 12606543

[73] Kovacs JJ, Murphy PJ, Gaillard S, Zhao X, Wu JT, Nicchitta CV, et al HDAC6 regulates Hsp90 acetylation and chaperone-dependent activation of glucocorticoid receptor. Mol. Cell. 2005;18:601–607. 10.1016/j.molcel.2005.04.021 15916966

[74] Geng H, Harvey CT, Pittsenbarger J, Liu Q, Beer TM, Xue C, et al HDAC4 protein regulates HIF1α protein lysine acetylation and cancer cell response to hypoxia. J Biol Chem. 2011;286:38095–38102. 10.1074/jbc.M111.257055 21917920 PMC3207467

[75] Ruchko MV, Gorodnya OM, Pastukh VM, Swiger BM, Middleton NS, Wilson GL, et al Hypoxia-induced oxidative base modifications in the VEGF hypoxia-response element are associated with transcriptionally active nucleosomes. Free Radic Biol Med. 2009;46:352–359. 10.1016/j.freeradbiomed.2008.09.038 18992807 PMC2645035

[76] Gao G, Li Y, Gee S, Dudley A, Fant J, Crosson C, et al Down-regulation of vascular endothelial growth factor and up-regulation of pigment epithelium-derived factor: a possible mechanism for the anti-angiogenic activity of plasminogen kringle 5. J Biol Chem. 2002;277:9492–7. 10.1074/jbc.M108004200 11782462

[77] Zhang SX, Wang JJ, Gao G, Parke K, Ma JX. Pigment epithelium-derived factor downregulates vascular endothelial growth factor (VEGF) expression and inhibits VEGF-VEGF receptor 2 binding in diabetic retinopathy. J Mol Endocrinol. 2006;37:1–12. 10.1677/jme.1.02008 16901919

[78] Qian DZ, Wang X, Kachhap SK, Kato Y, Wei Y, Zhang L, et al The histone deacetylase inhibitor NVP-LAQ824 inhibits angiogenesis and has a greater antitumor effect in combination with the vascular endothelial growth factor receptor tyrosine kinase inhibitor PTK787/ZK222584. Cancer Res. 2004;64:6626–6634. 10.1158/0008-5472.CAN-04-0540 15374977

[79] Kim JY, Hwang JH, Zhou W, Shin J, Noh SM, Song IS, et al The expression of VEGF receptor genes is concurrently influenced by epigenetic gene silencing of the genes and VEGF activation. Epigenetics 2009;4:313–321. 19633424

[80] Rubinfeld H, Seger R. The ERK cascade: a prototype of MAPK signaling. Mol Biotechnol. 2005;31:151–174. 10.1385/MB:31:2:151 16170216

[81] Franke TF, Kaplan DR, Cantley LC. PI3K: downstream AKTion blocks apoptosis. Cell 1997;88:435–437. pii: S0092-8674(00)81883-8 9038334 10.1016/s0092-8674(00)81883-8

[82] Gesbert F, Sellers WR, Signoretti S, Loda M, Griffin JD. BCR/ABL regulates expression of the cyclin-dependent kinase inhibitor p27Kip1 through the phosphatidylinositol 3-Kinase/AKT pathway. J Biol Chem. 2000;275:39223–39230. 10.1074/jbc.M007291200 11010972

[83] Huang WC, Chen CC. Akt phosphorylation of p300 at Ser-1834 is essential for its histone acetyltransferase and transcriptional activity. Mol Cell Biol. 2005;25:6592–6602. 10.1128/MCB.25.15.6592-6602.2005 16024795 PMC1190347

[84] Sang N, Stiehl DP, Bohensky J, Leshchinsky I, Srinivas V, Caro J. MAPK signaling up-regulates the activity of hypoxia-inducible factors by its effects on p300. J Biol Chem. 2003;278:14013–14019. 10.1074/jbc.M209702200 12588875 PMC4518846

[85] Wang QE, Han C, Zhao R, Wani G, Zhu Q, Gong L, et al p38 MAPK- and Akt-mediated p300 phosphorylation regulates its degradation to facilitate nucleotide excision repair. Nucleic Acid Res. 2012;41:1722–1733. 10.1093/nar/gks1312 23275565 PMC3561975

[86] Nicholson DW, Ali A, Thornberry NA, Vaillancourt JP, Ding CK, Gallant M, et al Identification and inhibition of the ICE/CED-3 protease necessary for mammalian apoptosis. Nature 1995;376:37–43. 10.1038/376037a0 7596430

[87] Shibuya M, Claesson-Welsh L. Signal Transduction by VEGF receptors in regulation of angiogenesis and lymphangiogenesis. Exp Cell Res. 2006;312:549–560. 10.1136/bjo.2004.063602 16336962

[88] Bruderer M, Alini M, Stoddart ML. Role of HOXA9 and VEZF1 in endothelial biology. J Vasc Res. 2013;50:265–278. 10.1159/000353287 23921720

[89] Viemann D, Goebeler M, Schmid S, Nordhues U, Klimmek K, Sorg C, et al TNF induces distinct gene expression programs in microvascular and macrovascular human endothelial cells. J Leukoc Biol. 2006;80:174–85. 10.1189/jlb.0905530 16617158

[90] Browning AC, Gray T, Amoaku WM. Isolation, culture, and characterisation of human macular inner choroidal microvascularendothelial cells. Br J Ophthalmol. 2005;89:1343–7. 10.1136/bjo.2004.063602 16170129 PMC1772898

[91] Wang H., Wittchen E. S., Jiang Y., Ambati B., Grossniklaus H. E., Hartnett M. E. Upregulation of CCR3 by Age-Related Stresses Promotes Choroidal Endothelial Cell Migration via VEGF-Dependent and -Independent Signaling. Invest Ophthalmol Vis Sci. 2011;52:8271–8277. 10.1167/iovs.11-8230 21917937 PMC3208059

[92] Rössig L, Urbich C, Brühl T, Dernbach E, Heeschen C, Chavakis E, Sasaki K, Aicher D, Diehl F, Seeger F, Potente M, Aicher A, Zanetta L, et al Histone deacetylase activity is essential for the expression of HoxA9 and for endothelial commitment of progenitor cells. J Exp Med. 2005;201:1825–1835. 10.1084/jem.20042097 15928198 PMC2213253

[93] Nakamura T, Mori T, Tada S, Krajewski W, Rozovskaia T, Wassell R, et al ALL-1 is a histone methyltransferase that assembles a supercomplex of proteins involved in transcriptional regulation. Mol Cell. 2002;10:1113–1128. 10.1016/S1097-2765(02)00740-2 12453419

[94] Park JH, Kim SH, Choi MC, Lee J, Oh DY, Im SA, et al Class II histone deacetylases play pivotal roles in heat shock protein 90-mediated proteasomal degradation of vascular endothelial growth factor receptors. Biochem Biophys Res Commun. 2008;368:318–322. 10.1016/j.bbrc.2008.01.056 18211808

[95] Dutta D, Ray S, Home P, Saha B, Wang S, Sheibani N, et al Regulation of angiogenesis by histone chaperone HIRA-mediated incorporation of lysine 56-acetylated histone H3.3 at chromatin domains of endothelial genes. J Biol Chem. 2010;285:41567–41577. 10.1074/jbc.M110.190025 21041298 PMC3009884

[96] Bhutto I, Lutty G. Understanding age-related macular degeneration (AMD): relationships between the photoreceptors/retinal pigment epithelium/Bruch’s membrane/choriocapillaris complex. Mol Aspects Med. 2012;33:295–317. 10.1016/j.mam.2012.04.005 22542780 PMC3392421

[97] Hirami Y, Mandai M, Takahashi M, Teramukai S, Tada H, Yoshimura N. Association of clinical characteristics with disease subtypes, initial visual acuity, and visual prognosis in neovascular age-related macular degeneration. Jpn J Ophthalmol. 2009;53:396–407. 10.1007/s10384-009-0669-4 19763758

[98] Qin L, Han YP. Epigenetic repression of matrix metalloproteinases in myofibroblastic hepatic stellate cells through histone deacetylase 4: implication in tissue fibrosis. Am J Pathol. 2010;177:1915–1928. 10.2353/ajpath.2010.100011 20847282 PMC2947286

[99] Tandon A, Tovey JC, Waggoner MR, Sharma A, Cowden JW, Gibson DJ, et al Vorinostat: a potent agent to prevent and treat laser-induced corneal haze. J Refract Surg. 2012;28:285–90. 10.3928/1081597X-20120210-01 22386369 PMC4508025

